# Hidden transport phenomena in an ultraclean correlated metal

**DOI:** 10.1038/s41467-024-48043-4

**Published:** 2024-06-24

**Authors:** Matthew Brahlek, Joseph D. Roth, Lei Zhang, Megan Briggeman, Patrick Irvin, Jason Lapano, Jeremy Levy, Turan Birol, Roman Engel-Herbert

**Affiliations:** 1 Department of Materials Science and Engineering, Pennsylvania State University, University Park, PA 16802 USA; 2 Materials Science and Technology Division, Oak Ridge National Laboratory, Oak Ridge, TN 37930 USA; 3 Department of Physics and Astronomy, University of Pittsburgh, Pittsburgh, PA 15260 USA; 4 Pittsburgh Quantum Institute, Pittsburgh, PA 15260 USA; 5 Department of Chemical Engineering and Materials Science, University of Minnesota, Minneapolis, MN 55455 USA; 6 Department of Physics, Pennsylvania State University, University Park, PA 16802 USA; 7 Department of Chemistry, Pennsylvania State University, University Park, PA 16802 USA; 8 Paul-Drude-Institut für Festkörperelektronik, Leibniz Institut im Forschungsverbund Berlin eV., Hausvogteiplatz 5-7, 10117 Berlin, Germany

**Keywords:** Electronic properties and materials, Surfaces, interfaces and thin films

## Abstract

Advancements in materials synthesis have been key to unveil the quantum nature of electronic properties in solids by providing experimental reference points for a correct theoretical description. Here, we report hidden transport phenomena emerging in the ultraclean limit of the archetypical correlated electron system SrVO_3_. The low temperature, low magnetic field transport was found to be dominated by anisotropic scattering, whereas, at high temperature, we find a yet undiscovered phase that exhibits clear deviations from the expected Landau Fermi liquid, which is reminiscent of strange-metal physics in materials on the verge of a Mott transition. Further, the high sample purity enabled accessing the high magnetic field transport regime at low temperature, which revealed an anomalously high Hall coefficient. Taken with the strong anisotropic scattering, this presents a more complex picture of SrVO_3_ that deviates from a simple Landau Fermi liquid. These hidden transport anomalies observed in the ultraclean limit prompt a theoretical reexamination of this canonical correlated electron system beyond the Landau Fermi liquid paradigm, and more generally serves as an experimental basis to refine theoretical methods to capture such nontrivial experimental consequences emerging in correlated electron systems.

## Abstract (web summary)

A correlated material SrVO_3_ has been considered to be a Fermi liquid, however previous studies have been limited to disordered samples. Here the authors study transport in ultraclean films of SrVO_3_, finding deviations from the Fermi liquid picture.

## Introduction

The comprehensive description of the electronic properties of solids is one of the great successes of quantum theory. At the most general level, this requires accounting for the kinetic and potential energy of electrons in the presence of atomic nuclei, as well as their mutual Coulomb interaction. Methods to describe the limiting case of a large electron kinetic energy relative to the electron-electron interaction, which justifies the use of a single determinant wavefunction. These have proven very successful and have delivered accurate predictions of electronic band structure and low energy excitation spectra of many material systems, provided that the ensemble of electrons can be recast as a weakly interacting electron gas. Continuous refinements to calculate the electronic ground state and band structure of materials from first principles utilizing modern computational methods have become a standard approach, now routinely achieving a very good agreement between theory and experiments^1–3^.

It has long been recognized that novel physics arises where the description of weakly interacting quasiparticles breaks down^4^. Electronic properties that originate from a sizeable electron correlation strength^5,6^ play a key role in quantum materials^7–10^, yet recent observations in myriad materials^11–14^ point to anomalies regarding the transport properties of the constituent electrons and interactions that give rise to novel transport phenomena^15^. An early approach to capture the physics of strongly interacting electrons proposed by Landau is based on the assumption that the interaction can be adiabatically switched on^16^, which enables capturing the electron interaction effects by representing the electron as Landau quasiparticles with finite lifetime and renormalized physical properties, such as mass. Using the non-interacting quasiparticle states as an underlying reference system; the many-body interaction is captured by the self-energy of the quasiparticle, which can be interpreted as an energy and momentum-dependent distribution of single particle lifetimes. A fundamental consequence from Landau’s Fermi liquid model derived by Luttinger is that the number of occupied states, i.e., the volume enclosed by the Fermi surface, is invariant of the electron correlation strength present and solely determined by the electron density of the system^17^. A violation of Luttinger’s theorem, therefore, implies that the underlying physics is not properly captured by Landau’s Fermi liquid theory.

Peculiar phenomena and anomalies in solids that deviate from the Landau Fermi liquid picture have been experimentally observed and are commonly referred to as non-Fermi liquids^18^ or strange metals. Here, both phases exhibit scaling relations of thermodynamic measurements (resistivity, specific heat, etc.) that differ from predictions of the Landau Fermi liquid. These exotic phases are known to occur, for example, in the normal conducting state of high-*T*_*C*_ superconductors^19^, which have long resisted a generally accepted explanation. A theoretical description that satisfactorily captures common features and explains these experimentally observed anomalies is key to unveiling and understanding the underlying physics in non-Landau Fermi liquids. These efforts have been complicated to some extent by the limits of materials quality commonly achieved in many systems exhibiting strong electron correlation, which is often driven by the fact that materials that exhibit correlation coincide with chemical complexity. In particular, stoichiometric control of the extensively studied *AB*O3 perovskite family has been found to be challenging because unintentional defects can easily form during synthesis^20^. As a result, these materials typically have a high defect concentration, potentially masking electron correlation effects.

Here, we present a detailed transport study of the archetypical correlated electron system SrVO_3_^21–26^ in the ultraclean limit. SrVO_3_ is an ideal testbed material for refining theoretical frameworks and improving analytic and numerical techniques since it is one of the simplest correlated metal systems. Specifically, it has a simple cubic structure, single electron occupancy of the *d*-orbitals, relatively weak but yet sizeable renormalization, and the absence of more complex phenomena, such as magnetic order or superconductivity^27^. Yet, experimental studies characterizing the spectroscopic and electronic transport properties of SrVO_3_ have been limited to materials with a high degree of disorder, manifested in relatively small residual resistivity ratios^28–35^. In contrast to previous reports, the transport measurements made here on ultraclean SrVO_3_ films revealed hidden features that deviate from the expectation of a simple Landau Fermi liquid commonly assumed for SrVO_3_. This alludes to a much more complex situation of this simple correlated electron system. Specifically, characteristics consistent with strange-metal behavior, manifested in a linear increase of the inverse Hall coefficient with temperature, were observed in materials on the verge of a Mott transitions such as the high *T*_*C*_ cuprates around room temperature^36–38^. At low temperatures the high carrier mobilities allowed probing electronic transport in the high magnetic field limit, revealing that ultraclean SrVO_3_ had an anomalously high Hall coefficient suggesting a possible violation of the Luttinger count, a key deviation from a simple Landau Fermi liquid. Further, the temperature dependence of the Hall coefficient in the low-magnetic-field limit can only be brought into agreement with theory only if there existed a strong ***k***-dependent transport relaxation time, in contrast to the common theoretical framework. To understand this, a simple model was used to experimentally extract relative transport relaxation times, which differed by more than one order of magnitude across the Fermi surfaces. These exotic transport anomalies did not occur for SrVO_3_ samples with a sizeable defect concentration, which are found to be in good agreement with current theoretical predictions^39^.

## Results and discussion

**Fig. 1:**
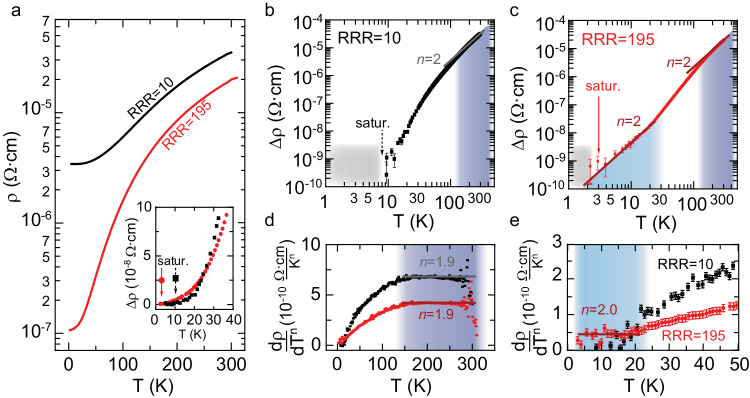
Temperature-dependent resistivity. **a** Resistivity *ρ* versus temperature for SrVO_3_ films in the disordered (black) and ultraclean (red) limit with residual resistivity ratios (RRR) of 10 and 195, respectively. Inset shows temperature-dependent resistivity difference *Δρ* = *ρ*(*T*)−*ρ*_0_ at low temperatures. The sample with low RRR already saturated at *T* = 10 K. Logarithmic plots of *Δρ* versus temperature for the **b**, **c**. disordered sample with RRR = 10 (**b**) and the ultraclean sample with RRR = 195 (**c**). For temperatures below the saturation value of *ρ*_0_, *Δρ* vanished. **d**, **e** Plots of the multiple resistivity scaling method $\frac{d\rho }{d\left({T}^{n}\right)}$ at **d** high temperature with *n* = 1.9 for the disordered (black) and ultraclean (red) limit, and (**e**) *n* = 2.0 at low temperatures in the ultraclean limit.

SrVO_3_ films studied here were grown by hybrid molecular beam epitaxy (*h*MBE), where the self-regulated growth kinetics enabled precise stoichiometry control^20,40,41^. As such, the *h*MBE growth technique was key to enable the comparison of films grown with low and high defect concentrations, which will be referred to as samples in the ultraclean and disordered limit. Transport measurements were performed on these samples using standard van der Pauw geometry as well as Hall bar structures, see Supplementary Note 1. The temperature-dependent resistivity for two representative samples of the ultraclean and disordered limit are shown in Fig. 1a with residual resistivities of *ρ*_0_ = *ρ*(2 K) ≈ 1  ×  10^−7^ Ω cm and 3 × 10^−6^ Ω cm, respectively. The room temperature resistivity was found to be quite similar for both samples with 2 × 10^−5^ Ω cm and 3 × 10^−5^ Ω cm, giving a high residual resistivity ratio RRR = *ρ*(300 K)/*ρ*(2 K) = 195 in the ultraclean, and a low RRR value of merely 10 in the disordered limit.

The dominant transport scattering mechanism in metals can be determined from the exponent *n* of the temperature-dependent resistivity *ρ*(*T*) = *ρ*_0_ + *A*_*n*_*·T*^*n*^, with *A*_*n*_ being a constant. Electron-phonon scattering gives rise to the Bloch–Grüneisen *T*^1^ and *T*^5^ scaling law of resistivity^42,43^, while an *n* = 2 exponent is caused by electron-electron scattering characteristic of a Fermi liquid and expected to occur well below temperatures at which phonons freeze out. Figure 1b, c shows the temperature-dependent change in resistivity *Δρ*(*T*) = *ρ*(*T*)−*ρ*_0_ across the entire temperature range in the *log-log* plot. Linear regimes in this representation provide direct access to extract the exponent *log*(*Δρ*(*T*)) = *n × log*(*T*) + *log*(*A*_*n*_). In the disordered limit, a quadratic dependence of resistivity (*n* = 2) was found at temperatures above 150 K, while at lower temperature the curve was nonlinear and saturated to the residual resistivity already at *T* = 10 K. In contrast, two linear regimes were found in the ultraclean limit, again one at temperatures above 150 K, and one below about 25 K. The fit values for the exponent *n* were confirmed using the multiple resistivity scaling method^44^ the derivative of resistivity with respect to *T*^*n*^ *was* plotted vs *T*. If the exponent *n* is correct then $\frac{d\rho }{d({T}^{n})}={A}_{n}$ is constant and temperature independent, see Fig. 1d, e. The latter method is more sensitive, and thus the high temperature regime with *T*^*2*^ dependence was refined to temperatures from (150 ± 20) K to 400 K (see Supplementary for additional data) for both the ultraclean and disordered limit. Remarkably, samples in the ultraclean limit also displayed a quadratic temperature dependence below (25 ± 5) K with *n* = 2 extracted from $\frac{d\rho }{d\left({T}^{n}\right)}$ plots shown in Fig. 1e. The experimental verification of electron scattering at low temperatures was made possible by the low degree of disorder and was hidden for disordered samples studied here, as well as in previous reports on SrVO_3_ films^28,30,45–47^.

**Fig. 2:**
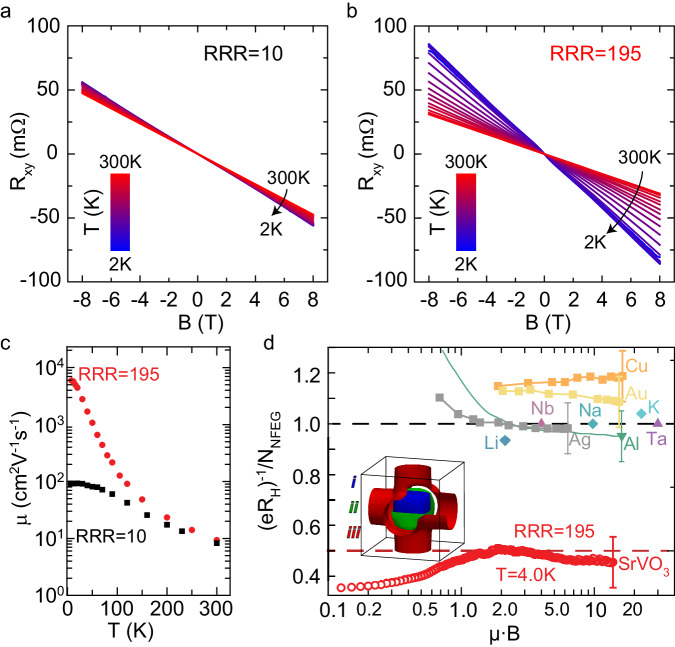
Temperature-dependent Hall effect and Hall coefficient in the high magnetic field limit. **a**, **b** Hall resistance R_xy_ versus magnetic field B and temperature T for SrVO_3_ films in the **a** disordered and **b** ultraclean limit. **c** Temperature-dependent mobility *μ* extracted from (A,B) for SrVO_3_ films in the disordered (RRR = 10, black squares) and ultraclean limit (RRR = 195, red circles). **d** Inverse Hall coefficient (*eR*_*H*_)^−1^ normalized to the carrier concentration *N*_NFEG_ of a nearly free electron gas in the high magnetic field limit *μ·B*»1 for selected metals from ref. ^48^ and ultraclean SrVO_3_. Maximum values *μ·B* = 1_3_.5 were achieved for SrVO_3_ in a magnetic field of 18.0 T having a carrier mobility of 7500 cm^2^ V^−1^ s^−1^. The inset shows the inner (i, blue), middle (ii, green), and outer (iii, red) Fermi surface sheets of SrVO_3_.

Temperature and magnetic field-dependent Hall effect measurements shown in Fig. 2a, b were performed on samples in the ultraclean and disordered limit to gain complementary insights into the physical processes that determine the transport in the correlated metal SrVO_3_. The negative slope of the Hall resistance *R*_*XY*_ indicated that electrons were the dominant carrier type, as expected for a single electron occupying the *3d t*_2g_ orbital forming the conduction band. The disordered sample showed a virtually temperature-independent linear Hall effect: the Hall coefficient *R*_*H*_ *=* *dR*_*xy*_*/dB* only slightly increased with decreasing temperature, see Fig. 2a. In contrast, the sample in the ultraclean limit showed a strong temperature dependence of the Hall coefficient along with the appearance of nonlinearity at relatively low magnetic fields, which will be discussed in detail below. Assuming a single carrier type the temperature-dependent electron mobility *μ* = *R*_*H*_*/ρ* was extracted and is shown in Fig. 2c. While the maximum carrier mobility in the disordered limit did not exceed 90 cm^2^ V^−1^ s^−1^, carrier mobilities reached values over 7500 cm^2^ V^−1^ s^−1^ in the ultraclean limit. This 80-fold increase enabled experimentally accessing the magneto-transport properties in the high magnetic field limit of SrVO_3_, i.e., where the product of carrier mobility *μ* and magnetic field strength *B* exceeded unity, i.e. *μ·B* > 1. This signifies that transport relaxation times are long enough that electrons can complete an orbit around the Fermi surface without scattering. Up until now, disorder in SrVO_3_ samples did not provide experimental access to this regime due to the low carrier mobility, which yielded values of *μ·B* < 0.2 in magnetic fields of ~18 T. In contrast, samples in the ultraclean limit achieved values *μ·B* ~ 14, well above the threshold.

If the magnetic field *B* is applied such that only closed orbits on the Fermi surface are possible then the Hall coefficient is not affected by the carrier scattering at such high fields. Rather, it is solely determined by the Fermi surface shape, and thus the carrier concentration^48^. Therefore, the carrier concentration *N*_*NFEG*_, calculated for a nearly free electron gas (NFEG) that accounts for the electronic band structure, can be directly compared to the measured value of the inverse Hall coefficient (*eR*_*H*_)^−1^ = *N*_*NFEG*_ with *e* being the elemental charge^48^. For conventional metals with rather simple Fermi surfaces, i.e. those which closely resemble a spherical shape small enough to be completely contained within the first Brillouin zone, the carrier concentration yields the number of valence electrons of the element. In the cases of Cu, Ag, Au, Li, Na, and K the inverse Hall coefficient gives *N*_*NFEG*_ ≈ 1 electron per primitive unit cell. Metals from elements with more than one valence electron exhibit much more complex Fermi surfaces, as they typically expand beyond the first Brillouin zone. In these cases, such as Al, Ta, and Nb, the calculations predict *N*_*NFEG*_ ≈ 1 hole per primitive unit cell, in excellent agreement with the experimental values of the inverse Hall coefficient (*eR*_*H*_)^−1^^48^.

The ratio of inverse Hall coefficient measured in high magnetic fields and carrier concentration *N*_*NFEG*_ are compiled in Fig. 2d for conventional metals with simple (Li, Na, and K), intermediate (Cu, Ag, and Au), and complex Fermi surface shapes (Al, Nb, and Ta), which were all found to converge to unity in the high magnetic field limit within experimental errors. In contrast, the inverse Hall coefficient of SrVO_3_ measured in the high magnetic field limit significantly differed from this conventional behavior. The ratio of (*eR*_*H*_)^−1^/*N*_*NFEG*_ = 0.47 ± 0.08 was much lower and saturated at less than half the expected value. As shown in Fig. 2d, a close inspection of the Fermi surfaces of SrVO_3_ (calculated by first-principles methods, as detailed in Supplementary Note 2) ruled out that this deviation was either from anomalous features of the Fermi surface geometry in SrVO_3_ or that trajectories on the Fermi surface were open, i.e., for a magnetic field applied normal to the film (***B*** \\ ***z***) all orbits were closed. The three different Fermi surface sheets derived from the *t*_*2g*_ bands were either spheroidal or topologically equivalent to Ta and Nb^49^, which both yielded (*eR*_*H*_)^−1^/*N*_*NFEG*_ ≈ 1^50^. Further, it is noted that the Hall coefficient is not affected by finite thickness in the high-field limit^51^. Thus, the measured deviation from the expected value is an intrinsic feature of the electronic properties of the correlated metal SrVO_3_, suggesting that the Luttinger count may be violated and SrVO_3_ deviates from a simple Landau Fermi liquid.

Another possible explanation of the anomalies of the high-field Hall coefficient would be that some of the itinerant carriers in ultraclean SrVO_3_ had anomalously short transport relaxation times such that they were not in the high magnetic field limit. If the carrier ensemble did not contribute to transport as a whole, transport relaxation times have to significantly depend on momentum, which is currently not reproduced by theory^52,53^. Elastic scattering time scales (those not representing momentum relaxing inelastic scattering events), such as the quasiparticle lifetime might be of relevance as well. Alternatively, as predicted by DMFT^1,26,39,54,55^ and confirmed in ARPES measurements^56–58^, the quasi-particle weight for SrVO_3_ is *Z*_*k*_ ≈ 0.5, which coincides very closely to the 50% discrepancy between carrier concentration of the nearly free electron gas and the measured Hall coefficient. Following this naive, but intuitive interpretation that the spectral weight of the incoherent side peaks of the quasiparticle are immobile, it could be speculated that in contrast to the predictions^5^, the Hall coefficient in the high magnetic field limit is renormalized by the value *Z*_*k*_. This is an interesting point for further theoretical investigation. Finally, no Shubnikov-de Haas oscillations were observed in any of the samples at high magnetic fields, as expected from the Fermi surface cross-sectional size determined from DFT analysis, for additional details see Supplementary Note 1d. The lack of oscillations suggests an anomalously low quantum scattering time that could be indicative of additional scattering mechanisms. Altogether, the experimental evidence that a more complex situation is present in correlated metals in the ultraclean limit is, therefore, far-reaching, and additional insight may be gained by examining time scales critical to correlated metals^59^.

**Fig. 3:**
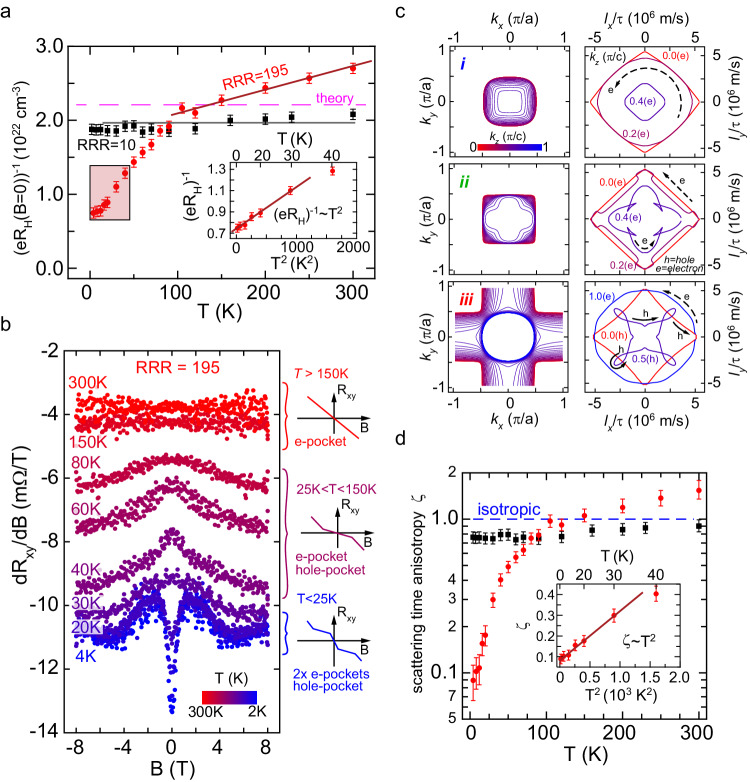
Hall coefficient in the low magnetic field limit and temperature-dependent nonlinear Hall effect. **a** Temperature dependence of the inverse Hall coefficient (eR_H_)^−1^ at low magnetic fields *B*→*0* for in the disordered (black squares) and ultraclean limit (red circles). The theoretically determined value assuming isotropic scattering is indicated as well. Inset shows *T*^2^ scaling behavior of the inverse Hall coefficient measured for ultraclean SrVO_3_ at low temperatures. **b** Slope of the Hall resistance *dR*_*xy*_*/dB* of ultraclean SrVO_3_ at different temperatures. Three schematic figures of the Hall resistance are shown on the right to categorize the type of multicarrier transport present: single electron (*T* > 150 K), electron and hole channel (25 K < *T* < 150 K), and two different electron and a single hole channel (*T* < 25 K), see details ref. **c** Hall coefficient analysis using the Fermi surface geometry. The three left panels show cross-sections of the different Fermi surface sheets *i*, *ii*, and *iii* at constant momentum values *k*_*z*_ representing the orbits on which electrons move in the presence of a magnetic field applied normal to the film plane. The right panels show the corresponding scattering space trajectories calculated from the cyclotron motion of the carriers. **d** Scattering time ratio $\zeta={\tau }_{o}/{\tau }_{i}$ determined for SrVO_3_ films in the disordered limit (black squares) and the ultraclean limit (red circles) by matching the inverse Hall coefficient determined from the Fermi surface geometry to the measured values, see Supplementary Note 3 for details. Inset shows *T*^2^ scaling behavior of the scattering time ratio $\zeta$ extracted for ultraclean SrVO_3_ at low temperatures.

Next, we focus on the Hall effect in the weak magnetic field limit *B*→0, which is sensitive to both the transport relaxation times as well as Fermi surface geometry^48,60^. Figure 3a shows the inverse Hall coefficient (*e·R*_*H*_)^−1^ in the temperature interval from 4 K to 300 K for the ultraclean and disordered limit (see Supplementary Note 1 for data to 400 K). Similar to previous reports^30^, the inverse Hall coefficient in the disordered limit had a value of 1.9 × 10^22 ^cm^−3^, which is in agreement with the nominal carrier concentration of 1.8 × 10^22 ^cm^−3^, as estimated by assuming one free electron per unit cell for SrVO_3_ with lattice parameter of 3.842 Å. Furthermore, (*e·R*_*H*_)^−1^ was temperature-independent, indicating that the temperature-dependent scattering mechanism due to electron-electron and electron-phonon interaction were masked by defect-induced scattering processes. In contrast, a strong temperature dependence was found in the ultraclean limit, where three different regimes could be distinguished: (1) A high-temperature regime above 100 ± 10 K where (*eR*_*H*_)^−1^ increased linearly with temperature, similar to cuprate high-*T*_*C*_ superconductors, which seemed to level off near 400 K (see Supplementary Note 1). (2) A transient regime between 30 to 100 K. (3) A low-temperature regime up to about 30 K where (*eR*_*H*_)^−1^ scaled quadratically with temperature (see inset, Fig. 3a). Furthermore, distinct nonlinearities of the Hall effect were present in the different regimes, which are shown in Fig. 3b by plotting the slope *dR*_*xy*_/*dB* for various temperatures. The nonlinearities can be interpreted as a signature that multiple carrier types with different mobilities contribute to transport, see detailed discussion in Supplementary Note 3. Above 100 K *dR*_*xy*_/*dB* was constant and negative, consistent with a single carrier type (electron-like). At lower temperatures, a relatively wide peak in *dR*_*xy*_/*dB* developed around *B* = 0 T. Here, the slope of the Hall resistance was shallower, consistent with the presence of two different carrier types, electron-like and hole-like. At temperatures between 40 and 30 K, the peak in *dR*_*xy*_/*dB* flattened at *B* = 0 T and a narrow dip occurred at temperatures below 20 K. While the slope was somewhat shallower at intermediate field strengths, it was much steeper at *B*→0 compared to the values at high magnetic fields, consistent with two distinguishable electron-like carriers having different mobilities and a single hole-like carrier with mobility in between the electron mobilities.

These experimental observations can be directly linked to the Fermi surface geometry of SrVO_3_ calculated by density functional theory, see Supplementary Note 2 for details. In the low magnetic field limit, the Hall coefficient was interpreted within the Jones-Zener solution of the Boltzmann transport equation^48,61^ using the elegant geometric approach developed by N. P. Ong^62^. This analysis enabled deciphering the complex factors affecting the low-field Hall coefficient, namely band structure (more specifically, Fermi surface curvature and Fermi velocity) and transport relaxation times. The three Fermi surface sheets arising from the *t*_*2g*_ bands—labeled *i*, *ii*, and *iii*—were decomposed into cross-sections stacked along the magnetic field direction. Circumferences of the cuts through the Fermi surface sheets along with their associated trajectories of the scattering path length vector in scattering space (***l***-space) were constructed for each Fermi surface cross section, as shown in Fig. 3c (see Supplementary Note 3 for more details). The scattering path length vectors ***l***_***k***_ were calculated from ***l***_***k***_ = *τ*_*k*_*·****v***_*F,****k***_, with ***k*** a Fermi wavevector, *τ*_***k***_ the ***k***-dependent transport relaxation time, and ***v***_*F,****k***_ the Fermi velocity given by ***v***_*F,****k***_ = ℏ^−1^***∇***_***k***_ *E*(***k***) with *E*(***k***) the band structure of SrVO_3_. The transport relaxation time was assumed to be a constant *τ*_***k***_ = *τ*, i.e., independent of the wavevector (isotropic). The inverse Hall coefficient can be calculated in the low-field limit by summing over the squares of the product of Fermi surface circumference and average scattering path length vector divided by the sum of the areas swept out by the trajectory of the scattering path length vector ***l***(***k***) in ***l***-space^62^, see Eq. 17 in the Supplementary Note 3. The handedness of the trajectory, along with the Fermi velocity determined whether the carriers, moving along the circumference of the Fermi surface sheets, have either electron or hole-like character. The analysis of the scattering space trajectories obtained from the Fermi surface sheets provided a qualitative explanation for the observed hole-like carriers that gave rise to the nonlinear Hall effect. The innermost Fermi surface sheets generated an electron-like response irrespective of the trajectories considered: all cuts of Fermi surface sheet *i* had positive curvature; the slightly larger Fermi surface sheet *ii* had segments with negative curvature from a dent-like feature along the <111> direction, ultimately generating an electron-like response because of the relative smaller Fermi velocity of these states. Nearly 50% of the Fermi surface sheet *iii* had hole-like character: while Fermi wavevector with *k*_*z*_ larger (smaller) than 0.5π/c (−0.5π/c) had a positive curvature, Fermi wavevectors with −0.5π/c <*k*_*z*_ < 0.5π/c had negative curvature, see Fig. 3c and Supplementary Fig. 7. Assuming a ***k***-independent, i.e., isotropic transport relaxation time, the low-field inverse Hall coefficient was solely dependent on the Fermi surface geometry. A value of (*eR*_*H*_)^−1^ = 2.2 × 10^22 ^cm^−3^ was obtained assuming a single transport relaxation time, in good agreement with the disordered limit throughout the entire temperature range (1.9 × 10^22 ^cm^−3^).

In the following we discuss the temperature dependence of the inverse Hall coefficient in the ultraclean limit. For conventional metals (*eR*_*H*_)^−1^ saturates to the isotropic scattering time at temperatures exceeding about 20% to 30% the Debye temperature *Θ*_*D*_, which is a consequence of the temperature dependence of the phonon population and magnitude of their wavevectors^48^. Above the Debye temperature, electrons can scatter to any position of the Fermi surface in the Brillouin zone, thus scattering times are averaged out and effectively become isotropic. As a result, the inverse Hall coefficient is solely determined by the Fermi surface geometry and thus becomes temperature independent^63^. SrVO_3_ has a Debye temperature of around 350 K^64,65^, implying that the transition to a temperature-independent (*eR*_*H*_)^−1^ should occur on the order of 100 K^63^. Instead, as shown in Fig. 3a, (*eR*_*H*_)^−1^ did not saturate in the ultraclean limit but increased linearly at temperatures higher than 100 K. This anomalous non-saturating behavior of (*eR*_*H*_)^−1^ coincides with the quadratic temperature dependence of resistivity, as shown in Fig. 1, providing further evidence that the dominant scattering mechanism in the high-temperature transport regime neither originates from electron-phonon nor electron-electron interaction, but likely stems from another exotic mechanism.

The quadratic temperature dependence of the inverse Hall coefficient at low temperatures is a signature of a strong ***k***-dependent scattering. While the low-temperature *T*^2^ dependence of the resistivity indicated electron-electron scattering as the dominant scattering mechanism, the same temperature behavior of (*eR*_*H*_)^−1^ further supported that a strong ***k***-dependence of transport relaxation times originated from a sizeable electron-electron scattering mechanism. In general, the scattering time depends continuously on ***k*** across the various Fermi surfaces. To gain initial insight into the experimental data and estimate the magnitude of variation in the scattering time, we can use a simple model that assigns a specific transport scattering time to each Fermi surface sheet. Here, an excellent agreement among the measured and calculated inverse Hall coefficients was found using Ong’s analysis throughout the entire temperature range^62,66,67^. The model utilized a unique scattering time *τ*_i_ for both inner Fermi surfaces *i* and *ii* with predominant electron-like character compared to the transport relaxation time *τ*_o_ for outer Fermi surface sheet *iii* with 50% hole character; this was motivated by the fact that the multicarrier analysis of the Hall effect which showed that electron- and hole-like portions of the Fermi surfaces had unique scattering times. The transport relaxation times were assumed constant over the entire Fermi surface sheet, and the transport relaxation time ratio *ζ* = *τ*_o_/*τ*_i_ was extracted as the only fitting parameter and is shown for the ultraclean and the disordered limit in Fig. 3d. The scattering was nearly isotropic for the disordered sample irrespective of temperature ($\zeta=1$), i.e., ${\tau }_{o}={\tau }_{i}$. An 11-fold decrease of the transport relaxation time *τ*_i_ of the two inner Fermi surface sheets *i* and *ii* relative to the transport relaxation time *τ*_*o*_ of the outer Fermi surface sheet *iii* was observed in the ultraclean limit. The transport relaxation time ratio $\zeta$ was found to scale with *T*^2^ up to about 30 K, as shown in the inset in Fig. 3d. This temperature dependence indicates the electron-electron interaction as the dominant scattering mechanism, which may be fundamentally anisotropic. This experimental result indicated a sizeable scattering time anisotropy to bring experimental values of (*eR*_*H*_)^−1^ into agreement with band structure calculations. This large difference in the transport relaxation times might provide an explanation for the violation of the Luttinger count: not all carriers are in the high magnetic field limit given the drastically different transport relaxation times extracted. However, current state-of-the-art theoretical models predict quasi-particle weight *Z*_*k*_ and electron-electron scattering rates to vary by less than 2% across the Fermi surface sheets of SrVO_3_^52,53^. First-principles calculations currently do not reproduce the intricacies of the electronic properties of the correlated metal SrVO_3_ observed in the ultraclean limit, but coincidentally agree with SrVO_3_ in the disordered limit.

The direct comparison of the ultraclean and the disordered samples revealed several hidden transport phenomena of the correlated metal SrVO_3_ that so far were masked by a sizeable defect concentration. In the ultraclean limit two different temperature ranges were distinguished in which the resistivity revealed a quadratic temperature dependence, separated by a transient regime. At high temperatures, a yet unobserved non-saturating behavior of the inverse Hall coefficient was discovered, while at low temperatures, a strong ***k***-dependent transport relaxation time was found. Hall effect measurements in the high-field limit revealed an apparent violation of the Luttinger count, suggesting that the SrVO_3_ ground state might be more complex than a simple Landau Fermi liquid, as generally assumed. The behavior near room temperature exhibits features reminiscent of strange metals. This contrasts with the expectation that carrier scattering by phonons dominates the electron transport behavior, which should give rise to a longitudinal resistivity following the Bloch–Grüneisen temperature dependence along with a saturation of the Hall coefficient. In contrast, a quadratic temperature dependence in the resistivity and a linear dependence in (*eR*_*H*_)^−1^ confirmed that the scattering near room temperature was not purely due to electron-phonon coupling. The high-*T*_*C*_ cuprate superconductors are the most famous example of strange metals in which the physics above the superconducting transition is still not well understood^15,68^. In light of the similarities of transport anomalies found in SrVO_3_ in the ultraclean limit with cuprates, and despite a different Fermi surface geometry and that electrons from the *t*_*2g*_ orbitals are involved. Therefore, it is possible that the physics of the normal-state in systems such as the high-*T*_*C*_ superconductors shares commonalities, and given that SrVO_3_ is theoretically tractable, this similarity may give new insight into this important problem.

Creating ever cleaner materials is a well-proven route to advance our understanding of the most basic electronic properties by enabling discovering new exotic electronic behavior and anomalies that cannot be explained within the existing theories. The transport phenomena observed in ultraclean SrVO_3_ samples provide new experimental insights into this archetypical material system exhibiting a sizeable electron correlation by revealing hidden phenomena, namely the presence of an anomalous strange-metal phase at room temperature, a pronounced ***k***-dependence of the electron scattering rate and an apparent violation of Luttinger’s theorem. The subtleties of these apparent deviations from a simple Landau Fermi liquid state were previously masked by defects. It appears that these defects alter the material responses such that coincidentally, a good agreement was found with theoretical predictions made within the framework of DFT and basic (single-site, etc.) DMFT, which causes SrVO_3_ to appear as a simple correlated metal. These experimental insights have dramatic consequences for the field of electron correlation, since SrVO_3_ has been widely accepted as canonical example of a correlated metal and has become a testbed material system to benchmark new theoretical methods and advanced models to capture the intrinsic nature of strongly interacting electron systems.^23,25,26,54,69–74^ The hidden transport phenomena unveiled in ultraclean samples will instigate a rethinking and expansion of the current theory describing electron correlation effects to clarify the origin of these phenomena, which will help decipher the underlying physics observed in these systems.

## Methods

Additional information, data, discussions, and the experimental (growth and transport) and theoretical (first-principles calculations and analysis of Hall measurements) methods can be found in the supplementary information file that includes references^20,21,39–42,48,62–65,75–81^.

### Supplementary information

Supplementary Information

## Data Availability

All data needed to evaluate the conclusions are present in the paper and supplementary materials. Additional data are available from the corresponding author upon reasonable request.
