# Supplementary material for: Hidden transport phenomena in an ultraclean correlated metal
Source: Nat Commun. 2024 Jun 24;15:5304. doi: 10.1038/s41467-024-48043-4 (PMC11196680; doi:10.1038/s41467-024-48043-4)
Supplement: Supplementary file 1 — Supplementary Information [file 41467_2024_48043_MOESM1_ESM.pdf]

Supplementary Information

Hidden transport phenomena in an ultraclean correlated metal

Matthew Brahlek[1,2⌧], Joseph D. Roth[1], Lei Zhang[1], Megan Briggeman[3,4], Patrick Irvin[3,4], Jason Lapano[1], Jeremy Levy[3,4], Turan Birol[5], Roman Engel-Herbert[1,6,7,8,*]

Affiliations:

[1]Department of Materials Science and Engineering, Pennsylvania State University, University Park, Pennsylvania 16802, U.S.A.
[2]Materials Science and Technology Division, Oak Ridge National Laboratory, Oak Ridge TN, 37930, U.S.A.
[3]Department of Physics and Astronomy, University of Pittsburgh, Pittsburgh, Pennsylvania 15260, U.S.A.

[4]Pittsburgh Quantum Institute, Pittsburgh, Pennsylvania 15260, U.S.A.

[5]Department of Chemical Engineering and Materials Science, University of Minnesota, Minneapolis 55455, U.S.A.
[6]Department of Physics, Pennsylvania State University, University Park, Pennsylvania 16802, U.S.A.
[7]Department of Chemistry, Pennsylvania State University, University Park, Pennsylvania 16802, U.S.A.
[8]Paul-Drude-Institut für Festkörperelektronik, Leibniz Institut im Forschungsverbund Berlin eV., Hausvogteiplatz 5-7, 10117 Berlin, Germany

Correspondence should be addressed to ⌧brahlekm@ornl.gov and *engel-herbert@pdi-berlin.de

1. **Experimental methods**
   a. **Growth of $SrVO_3$ by hybrid molecular beam epitaxy (*h*MBE)**
   b. **Magneto-transport measurements and data analysis**
   c. **Scaling analysis**
   d. **SdH analysis**
2. **First principles calculations of $SrVO_3$**
   a. **DFT and DMFT calculation details**
   b. **Analysis of Fermi surface sheets**
3. **The analysis of Hall measurements**
   a. **Nonlinear Hall effect from multicarrier conduction**
   b. **Geometric interpretation of the Hall coefficient in the weak magnetic field limit**
   c. **Temperature dependent Hall coefficient in $SrVO_3$**

1. **Experimental Methods**

a. Growth of $SrVO_3$ by hybrid molecular beam epitaxy (*h*MBE)

$SrVO_3$ films were grown using the hybrid molecular beam epitaxy (*h*MBE) technique. In contrast to conventional oxide MBE, which uses thermal evaporation of pure elements and the supply of oxygen or ozone, *h*MBE combines elemental sources with metalorganic sources. This combination is advantageous for the growth of multicomponent complex oxides such as the ABO_3 perovskites because the volatile character of the metalorganic allows accessing an adsorption-controlled growth regime. For films grown inside this growth window the cation ratio of the grown film is self-regulated, i.e. independent of the fluxes supplied to the sample. This growth mechanism enables the synthesis of highest quality perovskite oxide thin films[1] and has been instru mental to the realize the ultraclean correlated metal $SrVO_3$.

Following Refs. [2,3], the films in this work were grown using elemental Sr in combination with vanadium oxytriisopropoxide (VTIP). Sr was heated in a conventional effusion cell to ~430 °C. The exact cell temperature was set to the targeted Sr flux of 2.50×10^{13} atoms/($cm^2 s^1$), measured by a quartz crystal monitor located at the sample position prior to growth. The VTIP source was heated to 53 °C and connected via a heated gas inlet system to a gas injector to supply the VTIP molecules during film growth. The substrates used were 10×10 mm^2 (001) $(La_{0.3}Sr_{0.7})(Al_{0.65}Ta_{0.35})O_3$ (LSAT), which were cleaned in-situ prior to film growth by heating them up to 900 °C (thermocouple temperature of the substrate holder) and exposing them to an oxygen plasma at 250 Watts (reactor chamber background pressure 5×10^{-7} Torr during substrate clean). Film growth was performed at 900 °C with molecular oxygen at ~5×10^{-8} Torr. While the flux of Sr was held constant the pressure upstream of the VTIP gas injector, P_{VTIP}, was varied to map out the growth window. The growth window was determined by using the following three steps: (i) initial calibration was done using in-situ reflection high-energy electron-diffraction (RHEED)[1,4] to determine P_{VTIP} that gives the sharpest diffraction pattern free of secondary phases on the surface.[3] (ii) Ex-situ X-ray diffraction was used to determine the films with a minimum lattice parameter of 3.824 Å, which indicated a stoichiometric Sr:V ratio of 1:1.[2,3] (iii) Most importantly, low temperature resistivity was used to determine the highest quality samples which had the lowest residual resistivity.[2,3]

b. Magnetotransport measurements and data analysis

Transport measurements were performed from room temperature down to 2 K using Van der Pauw (VdP) and Hall bar geometries. For the ultraclean samples first VdP measurements were performed, followed by Hall bar measurements. Standard photolithography techniques followed by reactive ion etching was used to pattern Hall bars on the same sample. Transport measurements taken in both geometries agreed to within ~20%, which is within the expected error for the geometric correction used to convert raw resistance measurements to sheet resistance.[5]

Magnetotransport measurements performed on two separate systems, one installed at Penn State University with a maximum field of 8.0 T, and higher field data was obtained at the University of Pittsburg with a maximum field of 18.0 T. The measurements at Penn State were performed using a Quantum Design Physical Properties Measurement System (PPMS) in AC-mode with a source current of 100 μA. The VdP geometry was used with indium contacts pressed to the film. The data presented in Fig. 1, 2, and 3 of the paper were collected at Penn State. High magnetic field transport measurements were performed at the University of Pittsburg in a dilution refrigerator (Leiden MNK) with base temperature 20 mK. A transverse magnetic field applied in the direction normal to the film was varied between -18 T to +18 T by a liquid helium solenoid magnet (Oxford). To measure the magnetoresistance and Hall resistance, an AC voltage was sourced from a 24-bit DAC (NI 4461). Voltage amplitudes were set to either 10 mV or 30 mV and 13 Hz. Current, longitudinal voltage, and transverse voltage were amplified using differential amplifiers (Krohn-Hite 7008) before being digitized by a 24-bit ADC (NI 4461). Digitized signals were demodulated at 13 Hz using a software Lock-in amplifier. The data shown in Fig. 2 were collected in Pittsburgh.

The Hall effect data was anti-symmetrized numerically to eliminate the possible contribution of the longitudinal resistance, R_{xx}, into the Hall resistance R_{xy}. The Hall resistance is purely an odd function of magnetic field, while the longitudinal resistance is an even function,[6] which allows to separate the data. For a function $f(x)$ composed of odd and even parts, $f(x) = f_{odd}(x) + f_{even}(x)$, where $f_{odd}(-x) = -f_{odd}(x)$, and $f_{even}(-x) = f_{even}(x)$ the even and odd contribution can be determined from $f_{even}(x) = (f(x) + f(-x))/2$, and $f_{odd}(x) = (f(x) - f(-x))/2$, respectively. This procedure has been applied to the Hall resistance data using a numerical interpolation function and calculating $R_{xy,m}(-B_m)$, where $R_{xy,m}$ and B_m is the Hall resistance and the magnetic field measured. Then $R_{xy} = (R_{xy,m}(B_m) - R_{xy,m}(-B_m))/2$ is calculated to eliminate any even contribution of a longitudinal resistance R_{xx}. Supplement Fig. S1 shows both the measured and the anti-symmetrized Hall resistance $R_{xy,m}$ and R_{xy}, respectively.

Precise values for the sample thickness are required to calculate the carrier concentration form the sheet carrier concentration and to convert sheet resistance into resistivity, which accounts for the largest source of errors for bulk crystals.[7] Hall effect measurements provide the number of carriers per area, n_{2D}, which for a homogeneous material is simply the number of carriers per volume, N_{3D}, multiplied by the thickness, t, i.e. $N_{3D} = n_{2D} \times t$. Film resistivity was calculated from the sheet resistance (i.e. measured resistances multiplied by a geometric factor of $\pi/ln(2)$ for VdP geometry, or, for a Hall bar, the width divided by the separation of the leads, see Ref. [5]) multiplied by the film thickness. For the films studied here the thickness was extracted from X-ray diffraction using the Laue oscillations, see e.g. details in Ref. [3], as well as high-angle annular dark field scanning transmission electron microscopy (HAADF-STEM), shown in Suppl. Fig. S2. The thickness of the ultraclean sample was 63 nm, within 1-2 nm. The thickness of the disordered sample was determined to be 50 nm.[3] Unlike bulk samples, where the error in measurement geometry and thickness can be larger than 10%, epitaxial thin films have an error within about 2%.

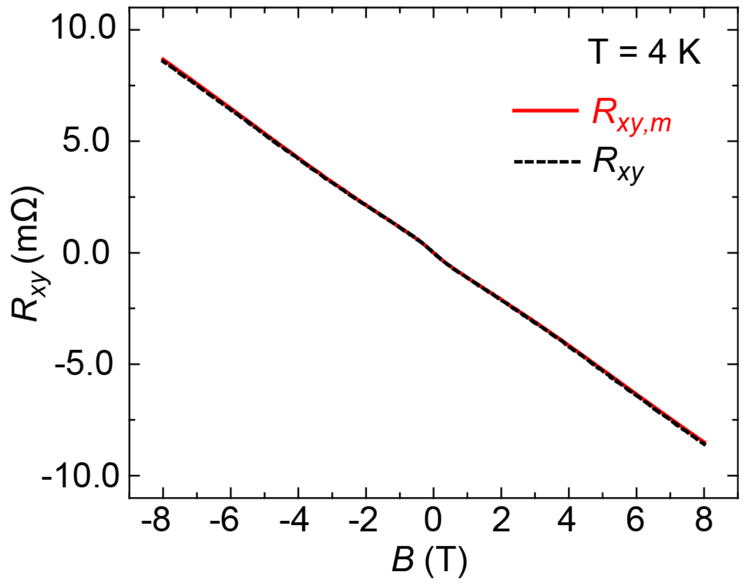

Fig. S1. Hall resistance R_{xy} as a function of magnetic field for $SrVO_3$ films in the ultraclean limit (RRR = 195). The measured data $R_{xy,m}$ is shown in red, while the odd contribution R_{xy} extracted from the data is shown as the black dashed curve.

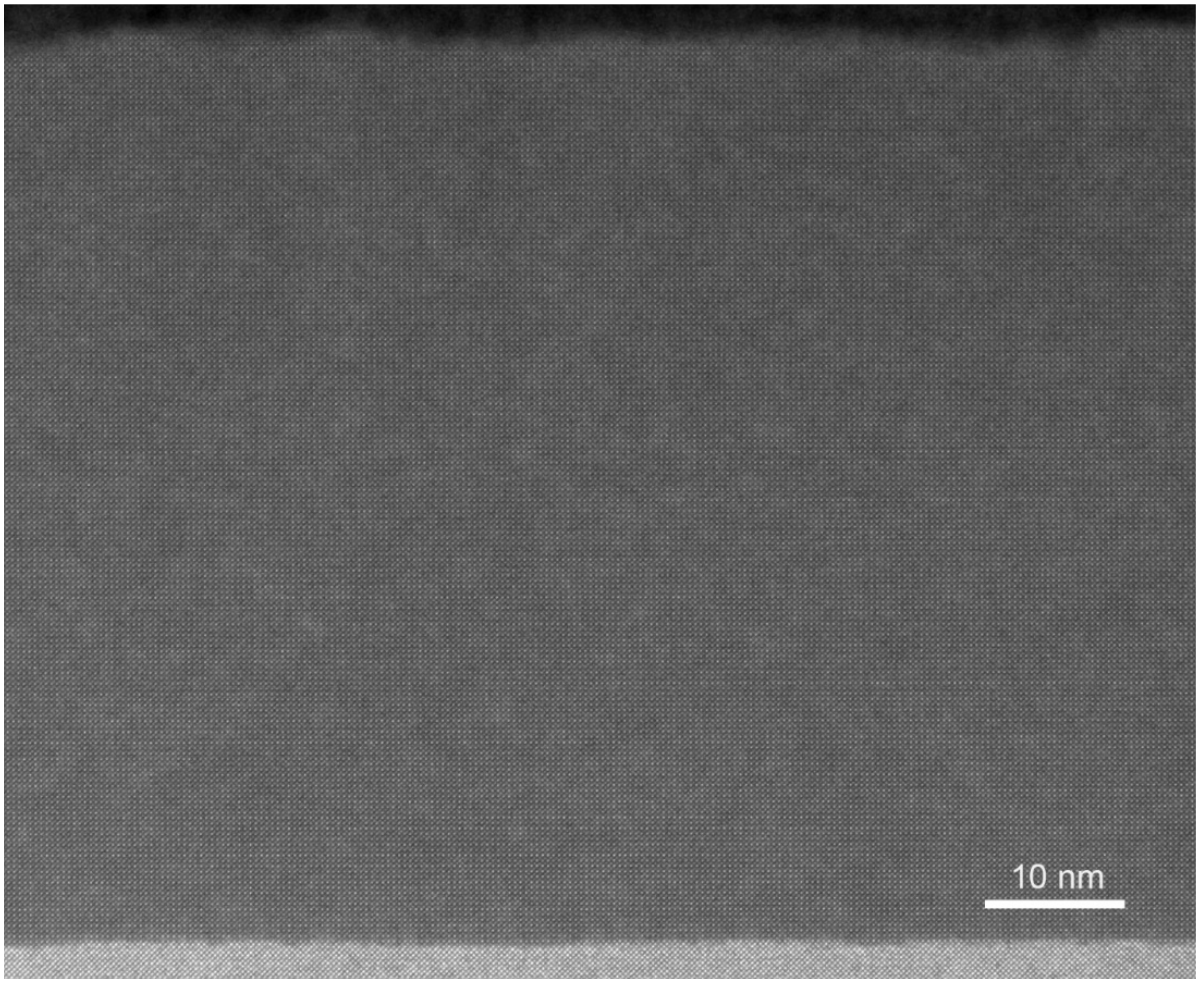

Fig. S2. High-angle annular dark field scanning transmission electron microscopy (HAADF-STEM) image of an ultraclean $SrVO_3$ film with thickness of 63 nm. See Ref. 8 for HAADF-STEM images of non-stoichiometric $SrVO_3$.

c. Scaling analysis

i. Simulated data to test analysis methods

We show in Fig. S3 a simulated data set where R~T^2 scaling was used between 0 to 100 K, T^1 between 100 and 200 K, and T^2, again between 200 to 400 K, see Fig. S3(a). This establishes that so long as $\frac{dR}{dT^n}$ is found to be constant with T, then the scaling relation is accurate over that regime. We connected these regions to match the R at 100 and 200 K as well as the derivatives of R such that the transition was perfectly smooth. Here, in the regime between 0 and 100K the function we used was $R(T) = 0.1T^2+100$, then the coefficients for the subsequent regions where calculated. We then smoothed the results, where the window of data points enabled introducing a ΔT transition (see Fig. S3(b) for dR/dT), as well as adding artificial noise (this was not found to affect the results for experimentally realistic levels). Shown in Fig. S3(c-d), the plots of R vs $T^{2.0}$ and $T^{2.2}$ yield semi quantitative confirmation of the power in the regions where T^2, which roughly confirms the scaling. This indicates that the accuracy of this method is low (IE there is a clear deviation of the regions with T^1 vs T^2, but T^2 vs $T^{2.2}$ cannot be distinguished). In Fig. S3(e-f), we calculated $\frac{dR}{dT^n}$ and allowed n to vary between 0.8 and 2.2. In panel e we have used a ΔT of 30K and in panel f we used a ΔT of 70 K. By looking carefully at these plots, we can see that the power can be accurately found for the case of ΔT of 30K (e) for all regions, as highlighted by the shaded squares. In contrast, for ΔT of 70K (f), the scaling power can only be confirmed for the higher temperature T^2 where the scaling region is sufficiently larger than ΔT. This shows that the transition region (where the scaling power is ill-defined) can be a significant portion of the region (specifically, ±30 K on a 100 K regime is still resolvable). Moreover, this shows that if the $\frac{dR}{dT^n}$ curve is flat over the interval, then the choice of n is sufficiently accurate.

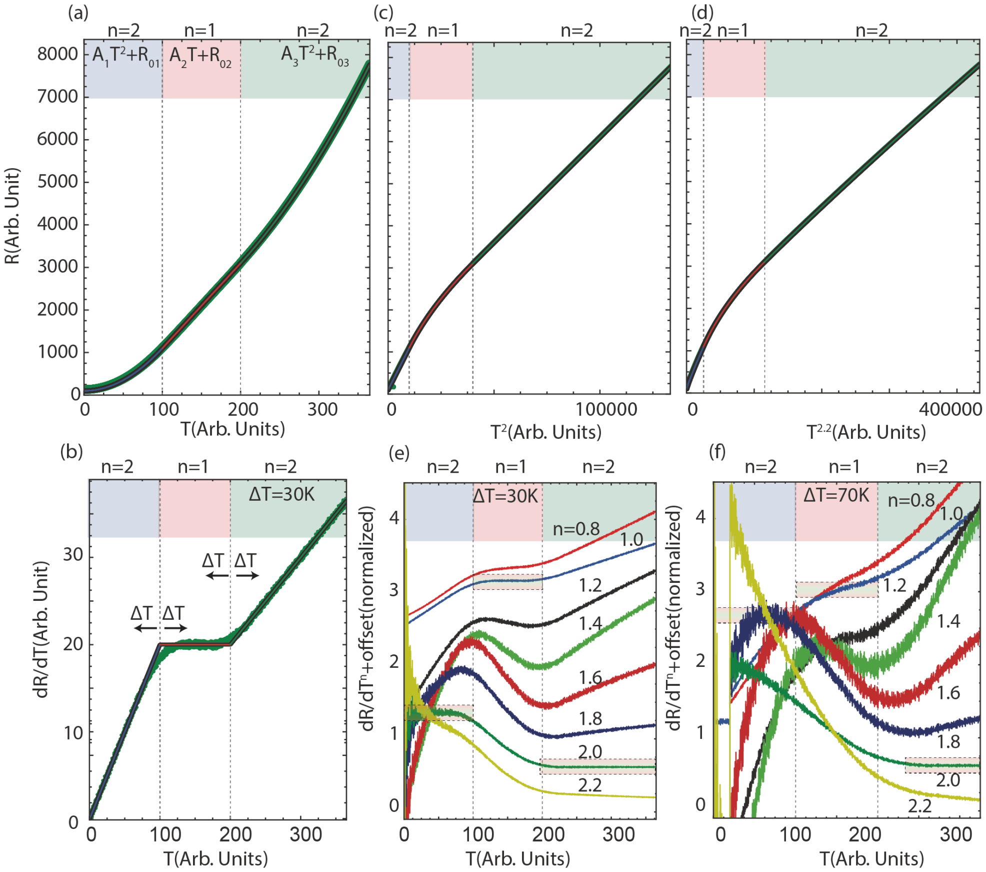

Fig. S3 Simulated training data for scaling analysis (a) $R\sim T^2$ scaling was used between 0 to 100 K (blue), T^1 between 100 and 200 K (pink), and, again, T^2 between 200 to 400 K (green). (b) plot of *dR/dT* with transitions among the region indicated as ΔT (here 30 K). (c-d) Plots of R vs T^2 (c) and $T^{2.2}$ (d). (e-f) Plots of dR/dT^n with n taken from 0.8 to 2.2 as indicated. The known powers are highlighted by the shaded box. These curves were normalized such that the max value is 1 and the minimum value is 0, then offset. The data is identical except (e) has ΔT = 30 K and (f) has ΔT = 70 K.

ii. Additional data (resistivity and Hall)

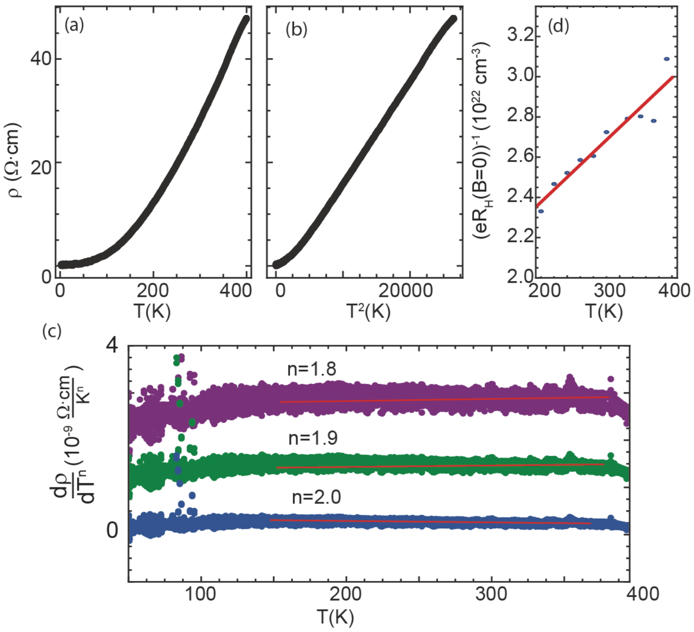

Fig. S4 (a-b) Temperature dependent resistivity *ρ(T)* of $SrVO_3$ with RRR=165 plotted (a) linear and (b) quadratic in temperature. (c) $d\rho/dT^n$ vs *T* plot for *n*=1.9, showing that the scaling law persist at higher than room temperature. (d) Temperature dependent carrier concentration deduced from Hall measurements. Red lines are guides to the eye.

d. SdH analysis

Despite a sufficiently high carrier mobility no Shubnikov-de Haas (SdH) oscillations were found, which are expected to occur from the Dingle analysis of quantum oscillations[8]. The magnetoresistance of $SrVO_3$ in the ultraclean limit is shown in Suppl. Fig. S5(a). The magnetic field step in these measurements was 7 mT, the raw data R_{xx} was smoothened using a 50-point average $R_{xx,ave}$ and a SdH magnetoresistance signal $\Delta R_{xx} = R_{xx} - R_{xx,ave}$ was calculated and is shown in Suppl. Fig. S5(b). No periodicity of ΔR_{xx} over 1/B was found, and the associated frequency spectrum shown in Suppl. Fig. S5(c) did not reveal any particular frequency components with amplitudes that were distinguishable against the background in the frequency range of 6 T to 18 T.

The SdH oscillations are periodic in B^{-1} and the associated SdH frequency Ω is related to the cross-section of the Fermi surface $A_{FS} = 2\pi \frac{e \cdot \Omega}{\hbar}$. The magnetic field difference $\Delta B = B_n - B_{n+1}$ between two adjacent SdH oscillations $\rho \sim \cos\left(\frac{2\pi \cdot \Omega}{B}\right)$ can be determined from the condition $\frac{2\pi \cdot \Omega}{B_n} = 2n \cdot \pi$ yielding a recursive formula to calculate B_{n+1} from B_n using $B_{n+1} = \frac{B_n \cdot \Omega}{B_n + \Omega}$. Estimates for the cross-sectional Fermi surface area for $SrVO_3$ obtained from typical Fermi vector lengths being about a quarter of a reciprocal lattice vector length $k_F \approx 0.39$ Å^{-1}, see DFT calculation of the Fermi surface sheet sizes, thus gave $\Omega = \frac{A_{FS} \cdot \hbar}{2\pi\ e} = 5005$ T. This value is in good agreement with the de Haas-van Alphen frequencies of 5 kT, 6 kT, and 9 kT measured on $CaVO_3$ single crystals reported in Ref.[9] .Using this frequency estimate of $\Omega = 5$ kT and typical magnetic field values available in the experiments ($B_n = 15$ T) a magnetic field difference between adjacent SdH oscillation maxima was $\Delta B \approx 45$ mT, about 6 times larger than the magnetic field steps used in the measurements. From this analysis we conclude that SdH oscillations could have been resolved in the experiment. Their absence indicated that the SdH amplitude was too small, which can be attributed to a low quantum mobility in $SrVO_3$. Elastic scattering dictates the SdH amplitude, which becomes small if the time between elastic scattering events is much shorter compared to the time between inelastic scattering events (momentum relaxation time). Another possible explanation could be a short quasiparticle lifetime in the system.

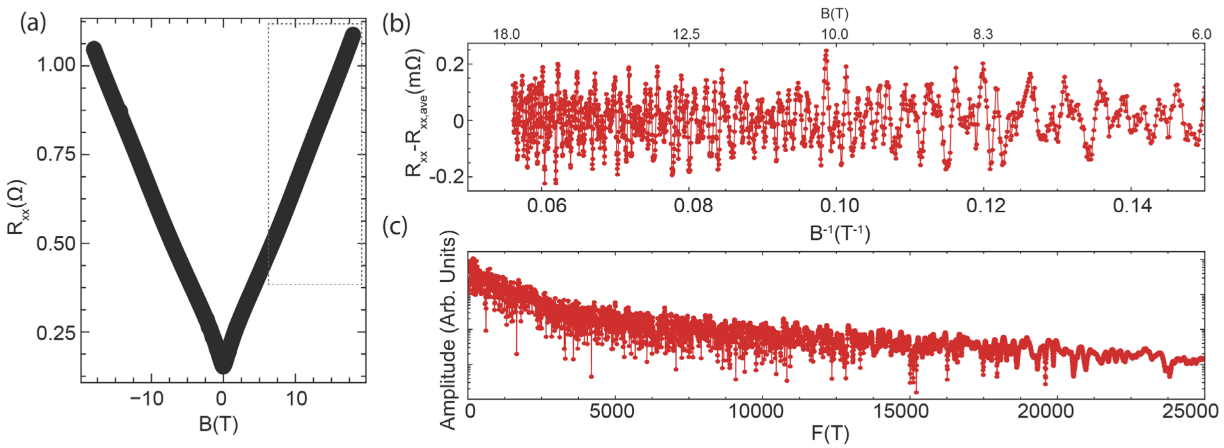

Fig. S5. (a) Magnetoresistance of ultraclean $SrVO_3$ taken at 4K The black curve R_{xx} represents the raw data, $R_{xx,ave}$ is the 50-point average. (b) Difference $\Delta R_{xx} = R_{xx} - R_{xx,ave}$ between raw data and 50-point average. No periodicity in ΔR_{xx} with $1/B$ is apparent. (c) Frequency spectra of ΔR_{xx} obtained by Fourier transform (FFT) data shown in (b).

2. First Principles Calculations of $SrVO_3$

First principles density functional theory (DFT) calculations were performed using Wien2K [P. Blaha, K. Schwarz, G. K. H. Madsen, D. Kvasnicka, and J. Luitz, WIEN2K: An Augmented Plane Wave+Local Orbitals Program for Calculating Crystal Properties (Technische Universität Wien, Austria, 2001)]. A 16×16×16 *k*-point grid was used for the primitive unit cell of $SrVO_3$, which consisted of 5 atoms. The radii of the atomic spheres for Sr, V, and O are 2.50, 1.85, and 1.70 Bohrs for Sr, V, and O, respectively. Fully-charge-self-consistent DFT+DMFT calculations were performed using the DMFT Wien2K EDMFTF package.[10] Onsite interaction parameters were picked as $U = 10$ eV and $J = 0.7$ eV. While the value of U was larger than, typical DFT+U implementations, the difference was due to the differences in the screening processes taken into account. These values have been previously shown to reproduce the angle-resolved photo-electron spectrum and optical properties.[11,12] The biaxial compressive strain of the $SrVO_3$ films has been taken into account by performing relaxations of the out-of-plane lattice parameter at fixed in-plane lattice parameter. Linear response calculations were used to confirm that no lattice instabilities emerged under biaxial strain. Self-energy obtained from the DMFT was analytically continued using the maximum entropy method and the quasiparticle weight Z was calculated from the slope of the real part of the self-energy. Despite breaking the cubic symmetry, the self-energy was found identical for the three t_{2g} orbitals within numerical error.

a. DFT and DMFT calculation details

The details of the band structure have been calculated and discussed in detail before[12] and are reproduced here for completeness. Supplement Fig. S6 shows the band dispersion of the t_{2g} band manifold intersecting the Fermi level resulting in three different Fermi sheets. The crystal field splitting of the t_{2g} and e_g orbitals is large enough resulting in a minimal band overlap. The lower lying oxygen *2p* bands form the valence band with a maximum about 2 eV below the Fermi level. The DFT+DMFT spectral function of $SrVO_3$ is shown side-by-side in Suppl. Fig. S6 on the same energy scale for easier comparison. The electronic correlation present on the vanadium site gives rise to a sizeable electronic self-energy resulting in a renormalized electron effective mass and hence reduced band width. The imaginary part of the self-energy at the Fermi energy is very small, resulting in sharp bands in the spectral function around the Fermi energy and a linear trend in the real part of the self-energy. The conduction band width is reduced by a

factor of Z ~ 0.55. From these calculation results a quasi-particle behavior well captured by the Landau Fermi liquid theory is expected.

b. Analysis of the Fermi surface sheets

$SrVO_3$ has three, non-intersecting Fermi surface sheets derived from the t_{2g} orbital manifold, see Suppl. Fig. S7(a). The outer, large Fermi surface sheet has a 'jungle gym' type structure where three cylindrical surfaces are perpendicular to one another. The middle and inner Fermi surfaces are both completely contained within the first Brillouin zone. While the middle Fermi surface can be approximated as cube-shaped sheet with 'faceted' corners and relatively sharp edges, the inner Fermi surface has more

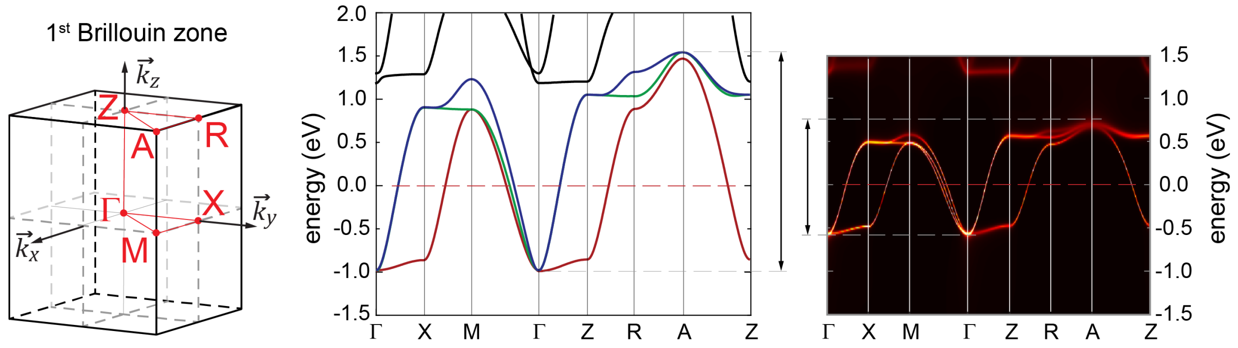

Fig. S6. First Brillouin zone (left) DFT band structure (middle) and DFT+DMFT spectral function (right) of $SrVO_3$. The tetragonal distortion is induced by the biaxial tensile strain reducing the out-of-plane lattice parameter along the z-axis.

'rounded' corners and edges. Their mutual distance and the distance to the outer Fermi surface is much reduced for wavevectors along the body diagonal (Γ→A) of the Brillouin zone. Fermi velocities are in the range of 3.7×10^7 m/s to 5.1×10^7 m/s for the jungle gym Fermi surface sheet, while somewhat larger velocities ranging up to 5.4×10^7 m/s were calculated for the two inner Fermi surface sheets, see Suppl. Fig.

S7(b). Note that again the lowest velocities are found for Fermi wave vectors pointing along the ⟨111⟩ direction. Aside from the *k*-dependent Fermi velocities the Fermi surface curvature directly affects the Hall coefficient in the weak magnetic field limit. Supplement Fig. S7(c) shows the *k*-dependent Fermi surface curvature of the individual sheets. The curvature is calculated for (k_x,k_y) orbits in the transport planes k_z=const. Note that a hole-type transport characteristic is displayed if the scattering length vector, i.e. the

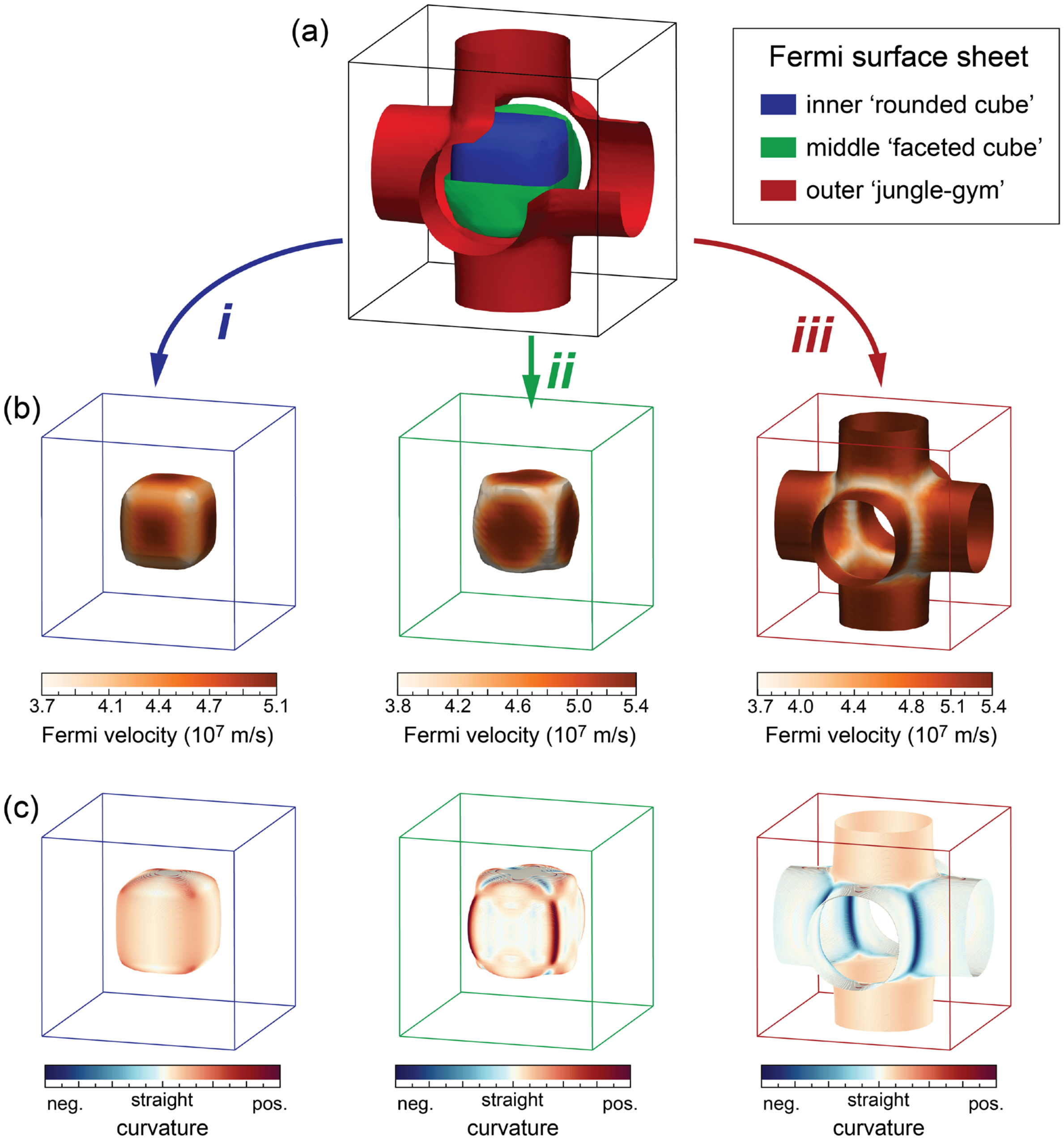

Fig. S7. (a) First Brillouin zone containing the three Fermi surface sheets of the trifold t_{2g} conduction bands of $SrVO_3$. Individual representation of the three Fermi surface with a color plot of (b) Fermi velocity and (c) Fermi surface curvature for Fermi surface orbits in the with k_z=constant.

product of Fermi velocity and transport relaxation time, is larger for Fermi surface regions with negative curvature compared to those with positive curvature, as will be discussed in detail in Suppl. Chapter 3b. This means that only the jungle-gym Fermi surface sheet can produce a sizeable hole contribution from Fermi surface orbits with negative curvature (blue shaded regions) and high Fermi velocity, which will occur in transport planes k_z=const. near the Γ point, i.e. for k_z values ranging from about $\frac{-\pi}{2\cdot c}$ to $\frac{\pi}{2\cdot c}$. While the inner Fermi surface sheet exhibit no negative curvature segments, the middle Fermi surface sheet has about 10% Fermi surface sheet orbits with negative curvature. These segments occur for electron wavevectors near the faceted corners, which have a much reduced Fermi velocity, thus exhibiting electron-like transport characteristics.

3. Analysis of Hall measurements

The analysis of the Hall measurement data is described in three parts. First the field dependence of the Hall coefficient $R_H=d\rho_{xy}/dB$ is quantitatively discussed to provide the necessary details for the interpretation of the nonlinear Hall effect arising from different carrier types present in $SrVO_3$. The presence of both electron and hole transport characteristics arises from the complexity of the Fermi surface shapes having segments with positive and negative curvatures and low and high Fermi velocities, respectively. A detailed description of the geometric interpretation of the Hall coefficient in the weak field limit is given. This approach has been proposed by N.P. Ong in Ref. [13] and is ideally suited to interpret the Hall data for 3D systems having multiple Fermi surface sheets with complex shapes, like $SrVO_3$. It is emphasized that this analysis is based on the assumptions that (1) the Boltzmann approach to describe the electronic transport is suitable, and (2) that magnetic skew scattering[14,15] is not present in $SrVO_3$. Finally, the analysis is applied to $SrVO_3$ and the temperature dependent nonlinearity of the Hall effect is explained.

a. Nonlinear Hall effect from multicarrier conduction

A homogeneous magnetic field imposes a force orthogonal to the direction of the velocity and applied magnetic field bringing the electrons onto a circular trajectory known as a cyclotron orbit. The electrons deflected by the magnetic field set up an electric field (Hall field E_H) in the direction transverse to both the current flow and magnetic field direction. The associated voltage difference in the transverse direction per unit current $I = j \cdot w \cdot t$ is referred to as the Hall resistance R_{xy}, with j, w and t being current density flowing along the x-axis, sample width (sample dimension along y-axis) and sample thickness (sample dimension along y-axis). While the first subscript indicates the direction of the current flow, the latter describes the transverse direction along which the voltage drop occurs. For electrons carrying the electric current the Hall resistance $R_{xy} = \frac{U_y}{I_x} = \frac{-E_H \cdot w}{j \cdot w \cdot t} = \frac{-v \cdot B \cdot w}{q \cdot N \cdot v \cdot w \cdot t} = -\frac{B}{q \cdot n_{2D}}$, with B the magnitude of the magnetic field, q the elemental charge, and n_{2D} the sheet carrier concentration, i.e. the number of carriers per unit area obtained by multiplying the 3D carrier density N with the sample thickness t.The Lorentz force and transverse electric field force acting on the electrons is equal $E_H = v \cdot B$. The Hall resistance R_{xy} is negative if negatively charged electrons conduct the electric current, because they are deflected in the -y-direction, thus setting up the transverse Hall field in the -y-direction. Conversely, if positive carriers carry the current the transverse Hall field is in the opposite, i.e. +y-direction, giving rise to a positive Hall

coefficient. For the simple case of either electron or hole conduction the Hall resistance is a linear function of the magnetic field with a slope inversely proportional to the sheet carrier concentration.

This result is classically derived by solving Newton's equation of motion for the carriers with effective mass m^* and charge q by describing the collisions between carriers with the atoms in the crystal by an average time τ between these collisions

$$m^* \left[\frac{d\vec{v}}{dt} + \frac{\vec{v}}{\tau}\right] = q \cdot \left(\vec{E} + \vec{v} \times \vec{B}\right) \qquad (1)$$

Further specifying the magnetic field to point along the z-axis $\vec{B} = (0 \quad 0 \quad B)$, confining the electric field driving the carriers to the x-y-plane $\vec{E} = (E_x \quad E_y \quad 0)$, and assuming an isotropic case, i.e. effective masses are independent of the transport direction, we find for the components of the drift velocity in the quasi-static case ($\frac{dv_x}{dt} = \frac{dv_y}{dt} = 0$), in which carriers move with constant drift velocity $\vec{v}_D = \begin{pmatrix} v_{Dx} \\ v_{Dy} \end{pmatrix}$ using the definition of carrier mobility $\mu = \frac{q \cdot \tau}{m}$

$$v_{Dx} = \frac{1}{1+\mu^2 \cdot B^2}\left[\mu \cdot E_x + \mu^2 \cdot B \cdot E_y\right] \qquad (2)$$

$$v_{Dy} = \frac{1}{1+\mu^2 \cdot B^2}\left[\mu \cdot E_y - \mu^2 \cdot B \cdot E_x\right] \qquad (3)$$

The electrical conductivity σ is a tensor quantity relating the current density $\vec{j} = q \cdot N \cdot \vec{v}_D$ generated by a carrier concentration *N* with individual charge q drifting in a steady-state motion with velocity $\vec{v}_D$ in the presence of an electric field $\vec{E}$ via $\vec{j} = \sigma \cdot \vec{E}$ with $\sigma = \begin{pmatrix} \sigma_{xx} & \sigma_{xy} \\ \sigma_{yx} & \sigma_{yy} \end{pmatrix}$. Relating the vector components of the current density j_i to the electric field components E_j via $j_i = \sigma_{ij} \cdot E_j$ using Eq. (2) and (3) gives

$$\sigma = \frac{q \cdot N}{1+\mu^2 \cdot B^2}\begin{pmatrix} \mu & \mu^2 \cdot B \\ -\mu^2 \cdot B & \mu \end{pmatrix} \qquad (4)$$

Since the tensor components of the resistivity tensor ρ are experimentally determined it is convenient to derive them by inverting the conductivity tensor σ

$$\rho = \sigma^{-1} = \frac{1}{\sigma_{xx} \cdot \sigma_{yy} - \sigma_{xy} \cdot \sigma_{yx}}\begin{pmatrix} \sigma_{yy} & -\sigma_{xy} \\ -\sigma_{yx} & \sigma_{xx} \end{pmatrix} = \begin{pmatrix} \rho_{xx} & \rho_{xy} \\ \rho_{yx} & \rho_{yy} \end{pmatrix} \qquad (5)$$

Simplifying the explicit expression of the individual resistivity tensor components we find for the longitudinal resistivity $\rho_{xx} = E_x / j_x$

$$\rho_{xx} = \frac{\sigma_{yy}}{\sigma_{xx} \cdot \sigma_{yy} - \sigma_{xy} \cdot \sigma_{yx}} = \frac{1+\mu^2 \cdot B^2}{q^2 \cdot N^2 \cdot \mu^2} \cdot \frac{q \cdot N \cdot \mu}{1+\mu^2 \cdot B^2} = \frac{1}{q \cdot N \cdot \mu} \qquad (6)$$

and for the transverse (Hall) resistivity $\rho_{xy} = E_y / j_x$

$$\rho_{xy} = \frac{-\sigma_{xy}}{\sigma_{xx} \cdot \sigma_{yy} - \sigma_{xy} \cdot \sigma_{yx}} = \frac{1+\mu^2 \cdot B^2}{q^2 \cdot N^2 \cdot \mu^2} \cdot \frac{-q \cdot N \cdot \mu^2 \cdot B}{1+\mu^2 \cdot B^2} = \frac{-B}{q \cdot N} \qquad (7)$$

From which the Hall coefficient is found

$$R_H = \frac{E_y}{j_x \cdot B} = \frac{\rho_{xy}}{B} = -\frac{1}{q \cdot N}$$

Nonlinearities in the Hall resistance $R_{xy} = \frac{\rho_{xy}}{t}$ (t: film thickness) arise if more than one carrier type contributes to transport. If the carrier motion can be assumed to take place in individual, non-interacting transport 'channels' distinguished by the index *i* with carrier densities N_i and mobilities μ_i for each distinct channel *i*. Now the conductivities of the individual channels simply add

$$\sigma_{tot} = \sum_i \sigma_i = \sum_i \frac{q \cdot N_i}{1+\mu_i^2 \cdot B^2}\begin{pmatrix} \mu_i & \mu_i^2 \cdot B \\ -\mu_i^2 \cdot B & \mu_i \end{pmatrix} \qquad (8)$$

and for the off-diagonal elements of the resistivity tensor follows

$$\rho_{xy} = -\frac{B}{q}\frac{\sum_i \frac{N_i \cdot \mu_i^2}{1+\mu_i^2 B^2}}{\left(\sum_i \frac{N_i \cdot \mu_i}{1+\mu_i^2 B^2}\right)^2 + \left(\sum_i \frac{N_i \cdot \mu_i^2 \cdot B}{1+\mu_i^2 B^2}\right)^2} \qquad (9)$$

The sign conversion of the Hall coefficient is accounted for by choosing a negative carrier concentration for channels with positively charged carriers.

To understand the qualitative behavior of the nonlinear Hall coefficient $R_H = dR_{xy}/dB$ with $R_{xy} = \rho_{xy}/t$ (t: film thickness) shown in Fig. 3(B) of the main manuscript we can take the above expression for the Hall resistivity in the limit of weak magnetic fields ($B \to 0$) as well as the strong magnetic field limit ($B \to \infty$; i.e. $B \gg \mu_i$). In the first case the second sum in the denominator is negligible and for the Hall resistivity in the weak magnetic field limit we find:

$$\rho_{xy,B\to 0} = -\frac{B}{q}\frac{\sum_i N_i \cdot \mu_i^2}{(\sum_i N_i \cdot \mu_i)^2} \qquad (10)$$

In strong magnetic fields the dominant term in the denominator is the second sum and the Hall resistivity in the strong magnetic field limit simplifies to:

$$\rho_{xy,B\to\infty} = -\frac{B}{q}\frac{1}{\sum_i N_i}. \qquad (11)$$

The Hall resistivity in the two limits are deliberately chosen to simplify the discussion how different carrier types give rise to different types of nonlinearities in the Hall effect. Since both resistivities in the weak and strong magnetic field limit $\rho_{xy,B\to 0}$ and $\rho_{xy,B\to\infty}$ vary linear with magnetic field B, the ratio $\rho_{xy,B\to 0}/\rho_{xy,B\to\infty}$, i.e. the ratio of the Hall resistivity slopes in the two limits, is independent of magnetic field. For the discussion the ratio $\rho_{xy,B\to 0}/\rho_{xy,B\to\infty}$ is calculated as function of the carrier mobility ratio μ_1/μ_2 and carrier concentration ratio N_1/N_2 of the respective channels, which is plotted in Suppl. Fig. S8. On the left side the carrier concentration of the two transport channels N_1 and N_2 have opposite sign, representing the case of mixed carrier types, where both electrons and holes are present. On the right side both channels have the same carrier type (electrons are assumed here); i.e. N_1 and N_2 have the same sign. The three panels below labeled (i), (ii) and (iii) show the Hall resistivity ρ_{xy} calculated from Eq. (9) as well as the slope of the Hall resistivity in the inset for three unique cases discussed below.

For the first case (two different carrier types) two different regions can be distinguished, defined for ratios $0<\rho_{xy,B\to 0}/\rho_{xy,B\to\infty}<1$ (yellow shaded) and $\rho_{xy,B\to 0}/\rho_{xy,B\to\infty}<0$ (brown shaded). In the first case the slope of the Hall resistivity is smaller as $B\to 0$ compared to the strong magnetic field limit. This type of nonlinearity typically occurs if more electrons ($|N_1/N_2|>1$) with higher mobilities ($\mu_1/\mu_2>1$) are present than holes. Further note that the same situation of a smaller slope of Hall resistivity at $B\to 0$ compared to the strong magnetic field limit also occurs for the case that there are more holes than electrons ($|N_1/N_2|<1$) with higher

hole mobility than electrons ($\mu_1/\mu_2<1$); however then the Hall resistivity has a positive slope. The case of a dominant electron channel with more carriers and a higher mobility is shown in panel (i) of Suppl. Fig. S8 for $|N_1/N_2|=2$ and $\mu_1/\mu_2=4$, which is similar to ultraclean $SrVO_3$ in the transient regime.

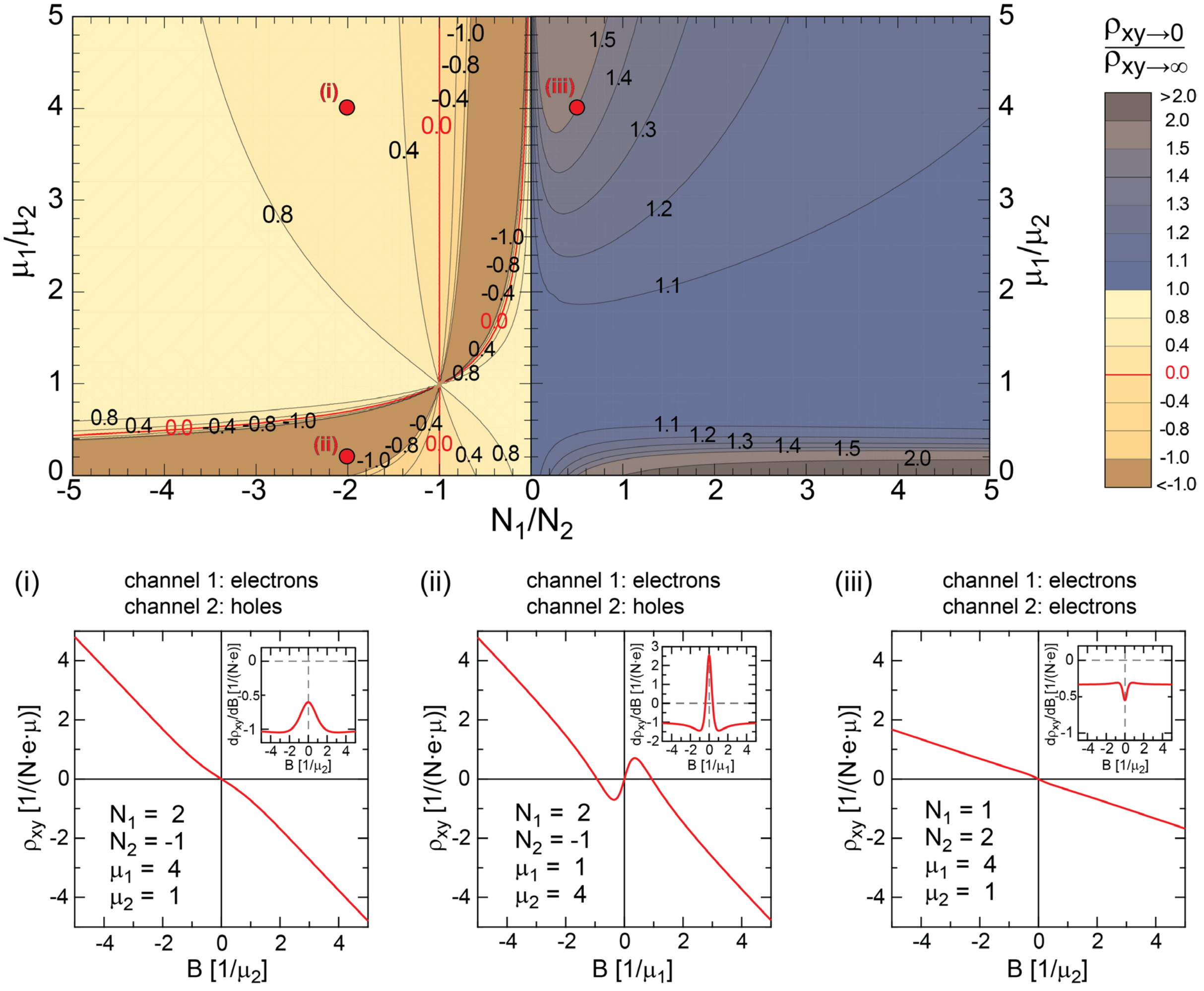

Fig. S8. Contour plot of the Hall resistivity ratio in the weak and strong magnetic field limit $\rho_{xy,B\to 0}/\rho_{xy,B\to\infty}$ plotted versus the ratio of carrier mobilities μ_1/μ_2 and carrier concentration N_1/N_2 of a two channel transport model. The left side of the plot is calculated for the case of electron-like and hole-like carriers and the contour $\rho_{xy,B\to 0}/\rho_{xy,B\to\infty} = 0$ is highlighted in red. The blue shaded right side of the graph is the ratio $\rho_{xy,B\to 0}/\rho_{xy,B\to\infty}$ calculated for two electron-like transport channels with different carrier concentration and mobilities. (i)-(iii) Calculated Hall resistivity as a function of magnetic field B, for different values of carrier concentrations N_1 and N_2 and carrier mobilities μ_1 and μ_2 of the respective transport channels. The respective insets show the change of the slope of the Hall resistivity $d\rho_{xy}/dB$ with magnetic field.

The second, brown shaded regions on the left side in Suppl. Fig. S8 the Hall resistivity ratio in the two field limits is negative: $\rho_{xy,B\rightarrow 0}/\rho_{xy,B\rightarrow\infty}<0$. This situation occurs if either the hole channel has much lower carrier concentration relative to the electron channel but a higher mobility ($|N_1/N_2|>1$ and $\mu_1/\mu_2<1$), or vice versa; i.e. the electron channel has much lower carrier concentration and higher mobility relative to the hole channel ($|N_1/N_2|<1$ and $\mu_1/\mu_2>1$). For the first case, e.g. specifically for $N_1/N_2=-2$ and $\mu_1/\mu_2=0.25$, the slope of the Hall resistivity is positive in the weak field limit and negative in the strong magnetic field limit, see middle panel (ii) in Suppl. Fig. S8. If instead the electron channel has lower carrier concentration and higher mobility, the slope of the Hall resistivity is negative in the weak field limit and positive in the strong magnetic field limit (not shown).

The nonlinear Hall resistivity regime shown in panel (iii) occurs if carriers in both transport channels are similar in type. In general, this scenario $N_1/N_2>0$ is shown on the right of Suppl. Fig. S8 (blue shaded area). Now the ratio $\rho_{xy,B\rightarrow 0}/\rho_{xy,B\rightarrow\infty} \geq 1$, i.e. the slope in the weak magnetic field limit is always steeper compared to the slope in the strong magnetic field limit. The panel (iii) depicts the specific case of $N_1/N_2=2$ and $\mu_1/\mu_2=4$.

If the simple model of non-interacting transport channels correctly captures the nonlinear Hall resistance effect the following categorization can be made:

- The slope of the Hall resistance in the weak field limit is steeper than in the strong field limit, $\rho_{xy,B\rightarrow 0}/\rho_{xy,B\rightarrow\infty} \geq 1$ [blue shaded region, example panel (iii)]: two channels with similar carrier type, but different mobilities and numbers of carriers within the respective channel are present. The degree of nonlinearity increases if the trend of carrier number ratio and mobility ratio of the respective channel are opposed, i.e. if $N_1/N_2<<1$ and $\mu_1/\mu_2>>1$ or $N_1/N_2>>1$ and $\mu_1/\mu_2<<1$, and the respective conductivity in the channel are comparable.
- The slope of the Hall resistance in the weak field limit is smaller than in the strong field limit. This always indicates a mixed carrier type, [yellow or brown shaded regions, example panels (i) and (ii)]. If the slopes in the weak and strong field limit have opposite signs the majority carrier type has a smaller carrier mobility [panel(ii)]. If electrons are the majority carrier type the slope in the weak field limit is positive. If the slopes in the weak and strong field limit have the same sign (panel (i)] then in most cases the majority carrier type has the higher carrier mobility. If electrons are the majority carrier type the overall slope is negative. (Exceptions can be found for very specific combinations of N_1, N_2, μ_1, and μ_2 that give rise to a positive ratio of the Hall resistance slopes in the weak and strong magnetic field limit $\rho_{xy,B\rightarrow 0}/\rho_{xy,B\rightarrow\infty} > 0$ which can be identified from Suppl. Fig. S8.

Within this context of multiband conduction, the observed nonlinearity of the Hall resistance for $SrVO_3$ shown in Fig. 3B of the main manuscript is attributed to the coexistence of electron-like and hole-like carriers, which are present due to positive and negative curvatures of the different Fermi surface sheets, see Suppl. Fig. S7. In the transient regime at intermediate temperatures between 25 K and 150 K the nonlinearity can be categorized as an electron and hole channel dominating the transport with the electrons being the majority carrier type having a higher mobility, while at low temperatures three transport channels can be distinguished, two electron-type channels with a low mobility and a high mobility, and a hole channel with intermediate mobility.

b. Geometric interpretation of the Hall coefficient in the weak magnetic field limit

A quantum mechanical description of transport beyond the simplified classical picture is required to properly account for electronic band structure effects of real materials and to ultimately derive a realistic formula for the longitudinal and transverse (Hall) resistivities that capture the material properties correctly. This is commonly approached by employing the Boltzmann transport equation. For systems with multiple Fermi surfaces and complex shapes the interpretation of the Hall effect in the weak magnetic field limit is highly non-trivial.[7] A more tangible and straight-forward geometric interpretation[13] invokes the representation of the carrier motion not only in reciprocal space (***k***-space), but also in 'scattering' space (***l***-space). Here, the 'scattering path length vector' $\boldsymbol{l}(\boldsymbol{k}) = \boldsymbol{l}_{\boldsymbol{k}} = \tau_k \cdot \boldsymbol{v}_{F,\boldsymbol{k}}$ of an electron with wavevector ***k*** is considered with $\tau_{\boldsymbol{k}}$ the transport relaxation time and $\boldsymbol{v}_{F,\boldsymbol{k}} = \hbar^{-1} \cdot \nabla_{\boldsymbol{k}} E(\boldsymbol{k})$ the Fermi velocity calculated from the band structure $E(\boldsymbol{k})$ with $\hbar$ the reduced Planck's constant. By transforming the Boltzmann transport equation from ***k***-space to ***l***-space the Hall conductivity $\sigma_{x,y}$ in the weak field limit can be expressed in 'simple' geometric terms using the trace of the scattering path length vector.[13] For a two-dimensional conductor with arbitrary Fermi surface shape the Hall conductivity $\sigma_{x,y}$ (in units of the quantized Hall conductance e^2/h, i.e. the inverse of the von Klitzing constant R_K) is equal to twice the magnetic flux quanta $\phi_0 = h/e$ of the field B going through the area A_l swept out by the scattering path length vector $\boldsymbol{l}(\boldsymbol{k})$ in ***l***-space:

$$\sigma_{xy} = \frac{2}{R_K} \cdot \frac{\phi}{\phi_0} = 2\frac{e^2}{h} \cdot \frac{\phi}{\phi_0} = 2\frac{e^2}{h} \cdot \frac{B \cdot A_l}{\phi_0} \qquad (11)$$

This expression of the 2D Hall conductivity can be generalized to three dimensions by integrating over the 'slices' of the Fermi surface in the transport plane that is normal to the magnetic field:[13]

$$\sigma_{xy}^{3D} = 2\frac{e^2}{h} \cdot \frac{B}{\phi_0} \int_{-\frac{\pi}{c}}^{\frac{\pi}{c}} A_{l_\perp}(k_z) \frac{dk_z}{2\pi} \qquad (12)$$

with c the out-of-plane lattice parameter. The subscript $l_\perp$ is the projection of the scattering length vector onto the transport plane perpendicular to the B-field.

Before specifying the equation used to calculate the Hall resistivity for 3D systems with multiple and complex Fermi surfaces – like the case of $SrVO_3$ – it is worth reviewing the key idea behind the geometric interpretation of the Hall effect in the weak magnetic field limit.[13] This elegant representation is particularly compelling because the complex factors affecting the Hall conductivity, namely band structure (more specifically Fermi surface curvature and Fermi velocity) as well as anisotropic scattering – i.e. a *k*-dependent transport relaxation time $\tau_{\boldsymbol{k}}$, can be separately discussed. Consider the carrier motion in ***k***-space in the presence of a magnetic field, where electrons sweep out orbits on the circumference of the Fermi surface in the plane normal to the magnetic field. For a spherical Fermi surface these ***k***-space orbits are circular, which under the assumption of a constant and isotropic transport relaxation time $\tau_{\boldsymbol{k}}$ translates to a similar, circular trace in ***l***-space: a main loop of the 'scattering path length vector' $\boldsymbol{l}_{\boldsymbol{k}} = \tau_k \cdot \boldsymbol{v}_{F,\boldsymbol{k}}$ enclosing the area A_e, as illustrated in Suppl. Fig. S9A. Note that the circular shape of the 'scattering path length vector' trace is because the Fermi velocity vector $\boldsymbol{v}_{F,\boldsymbol{k}}$ is parallel to the wavevector ***k*** for the simple case of a spherical Fermi surface.

Fermi surfaces of real materials, such as $SrVO_3$, can deviate substantially from a simple circular shape of a simple Fermi surface to the extent that it can contain segments with negative curvature, as schematically shown in Suppl. Fig. S9B. Here, the local curvature $\kappa = \frac{d\theta}{dk_t}$ defined as deviation from a

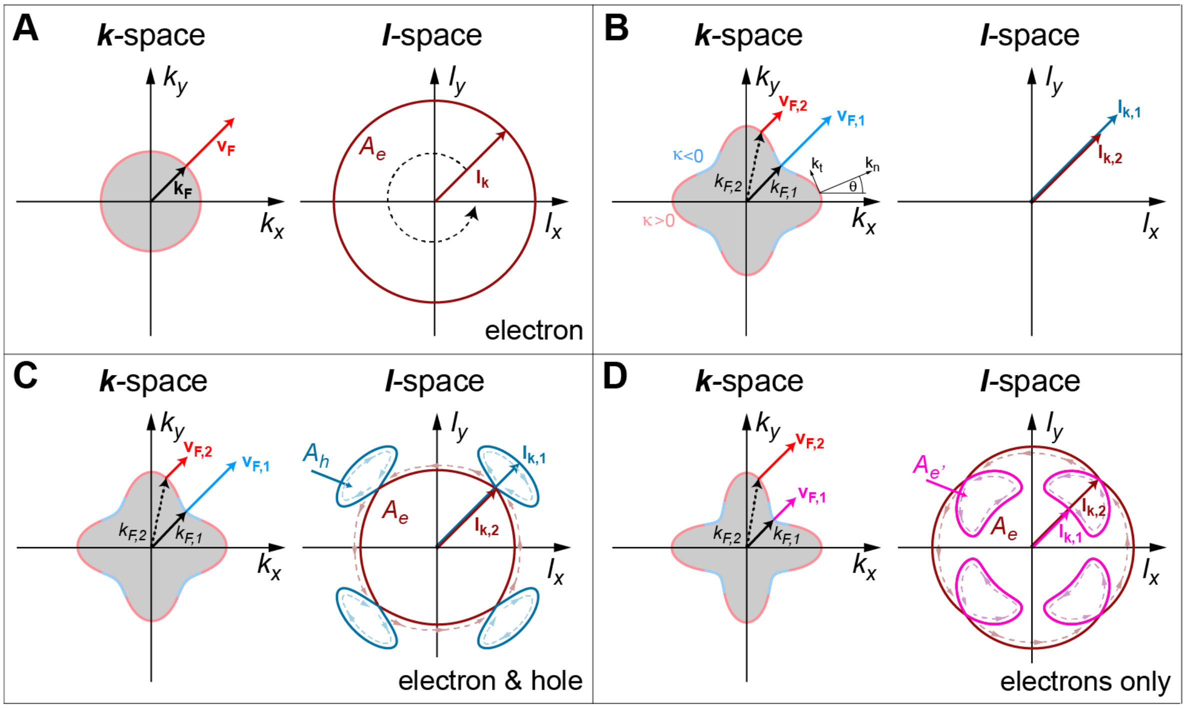

Fig. S9. Schematic of Fermi surfaces represented in ***k***-space (left) and traces of the scattering path length vector in ***l***-space (right). (**A**) Circular Fermi surface generates circular trace in ***l***-space. (**B**) Distorted Fermi surface giving rise to Fermi surface segments with negative curvature κ (blue). Fermi velocity vectors $v_{F,1}$ and $v_{F,2}$ associated to different Fermi wavevectors $k_{F,1}$ and $k_{F,2}$ can now be parallel. (**C**) Large Fermi velocities in Fermi surface segments with negative curvature that cause additional 'outer' loops with opposite handedness in ***l***-space and a mixed electron/hole carrier characteristic in the Hall conductivity σ_{xy}. (**D**) Fermi surface with more pronounced distortion as in (C) and a relative smaller Fermi velocity for states with negative curvature. A more pronounced distortion results in larger additional loops in ***l***-space, the smaller Fermi velocity causes 'inner loops' with same handedness of the main loop, resulting in electron-like transport characteristics only.

straight line $d\theta$ per arc-length dk_t (tangential) is inverse to the radius approximating the local bending of the Fermi surface. The presence of Fermi surface segments with negative curvature result in a nontrivial change in the scattering path length vector trajectory in ***l***-space: the emergence of additional loops in ***l***-space. Now, Fermi velocity vectors for different ***k***-states are parallel. Note that the Fermi velocities $\boldsymbol{v}_{F,1}$ and $\boldsymbol{v}_{F,2}$ in Suppl. Fig. S9B are parallel for two non-parallel Fermi wavevectors $\boldsymbol{k}_1$ and $\boldsymbol{k}_2$. Therefore, the corresponding scattering path length vectors $\boldsymbol{l}_{k,1}$ and $\boldsymbol{l}_{k,2}$ point in the same direction. Depending on the magnitude of the Fermi velocity and the transport relaxation time of the specific Fermi wavevectors $\boldsymbol{k}_1$ and $\boldsymbol{k}_2$ the scattering path length vectors $\boldsymbol{l}_{k,1}$ and $\boldsymbol{l}_{k,2}$ have different lengths. If ***k***-states on Fermi surface segments

with negative curvature have a large scattering path length vector, either due to a larger Fermi velocity or a longer transport relaxation time, the additional loops emerging in ***l***-space have opposite rotation sense compared to the main loop, see Suppl. Fig. S9C. Electrons undergo a retrograde motion, thus qualitatively behaving like positively charged carriers. Conversely, if ***k***-states on Fermi surface segments with negative curvature have a shorter scattering path length vector due to either a smaller Fermi velocity or a shorter transport relaxation time, the rotation sense of the additional loops is the same as the main loop in ***l***-space and carriers behave like electrons in transport irrespective of their ***k***-state, see Suppl. Fig. S9D.

The local Fermi surface curvature along with the Fermi velocity and transport relaxation time of their respective ***k***-states has direct consequences for the Hall conductivity σ_{xy} that is 'encoded' in A_l, see Eq. (11). For the simple case of the Fermi surface shown in Suppl. Fig. S9A with one main loop $\sigma_{xy} \sim A_e$, with A_e the area enclosed by the trace of the scattering path length vector in ***k***-space. For more complex Fermi surfaces shown in Suppl. Fig. S9C-D the Hall conductivity is proportional to the sum of all enclosed areas traced out by the scattering path length vector, where the handedness must be taken into account. Specifically, for the cases shown in Suppl. Fig. S9C Hall conductivity $\sigma_{xy} \sim (A_e - A_h)$. The larger the segments of negative Fermi surface curvature and the longer scattering path length vector the more pronounced the hole-like character of carriers on the Hall conductivity. This is intuitively clear, since for a larger Fermi velocity of these states and a longer transport relaxation time they can travel further between scattering events, thereby influencing the overall transport characteristics stronger. For the case shown in Suppl. Fig. S9D the smaller Fermi velocity for Fermi wavevectors with negative curvature resulted in additional loops in ***l***-space with same handedness which gives rise to a larger Hall conductivity $\sigma_{xy} \sim A_e + A_{e'}$ and the absence of hole-like character.

While the effect of Fermi surface curvature and Fermi velocity is exemplarily discussed for a 2D conductor, extension to a 3D Fermi surface is straightforward by summing over individual cross sections of the 3D Fermi surface (Eq. (12)). Further extension to multiple Fermi surfaces is done by summing over the individual Fermi surface sheets arising from the different bands.[13]

Similar to the discussion about multi-carrier transport effects it is convenient to derive the Hall resistivity from the Hall conductivity. Since $\sigma_{xy}, \sigma_{yx} \ll \sigma_{xx}, \sigma_{yy}$ in the weak magnetic field limit, as explicitly shown in (Eq. (9)), the Hall resistivity can be approximated as

$$\rho_{xy} = \frac{\sigma_{xy}}{\sigma_{xx} \cdot \sigma_{yy} - \sigma_{xy} \cdot \sigma_{yx}} \approx \frac{\sigma_{xy}}{\sigma_{xx} \cdot \sigma_{yy}} \qquad (13)$$

The longitudinal conductivity of a 2D conductor that takes the specific Fermi surface shape into account can be expressed as[13]

$$\sigma_{xx} = \frac{e^2}{h} \cdot \frac{l_{av} \cdot S}{2\pi} \qquad (14)$$

with S being the circumference of the Fermi surface orbit and l_{av} the average of the scattering path vector length of ***k***-states of that Fermi surface orbit

$$l_{av} = \int dk_t \frac{|\vec{l}|_{\vec{k}}}{S} \qquad (15)$$

Assuming a four-fold symmetry in the plane ($\sigma_{xx} = \sigma_{yy}$) – like in the case of biaxially strained $SrVO_3$ – the Hall coefficient $R_H = \frac{\rho_{xy}}{B}$ for a 2D conductor can be expressed using Eq. (13), (14) and (11)

$$R_H = \frac{\rho_{xy}}{B} = \frac{1}{B} \cdot \frac{\sigma_{xy}}{\sigma_{xx} \cdot \sigma_{yy}} = \frac{2\frac{e^2}{h \cdot \phi_0} \cdot A_l}{\left(\frac{e^2}{2\pi \cdot h} \cdot l_{av} \cdot S\right)^2} = \frac{8\pi^2}{e} \cdot \frac{A_l}{(l_{av} \cdot S)^2} \qquad (16)$$

Generalizing this expression to three dimensions by integrating over the 'slices' dk_z of the Fermi surface in the transport plane and considering all three Fermi surfaces indexed by the subscript j the inverse Hall coefficient can be expressed as

$$(eR_H)^{-1} = \frac{1}{8\pi^2} \cdot \frac{\left[\sum_j \int_{-\frac{\pi}{c}}^{\frac{\pi}{c}} l_{av,k_z,j} \cdot S_{k_z,j} \frac{dk_{z,j}}{2\pi}\right]^2}{\sum_j \int_{-\frac{\pi}{c}}^{\frac{\pi}{c}} A_{l,k_z,j} \frac{dk_{z,j}}{2\pi}} \qquad (17)$$

where $l_{av,k_z,j}$ is the average scattering path length vector of the scattering path vectors $\boldsymbol{l}_{\boldsymbol{k},k_z,j}$ linked to $\boldsymbol{k}$-states lying on the 'orbit' of constant $k_{z,j}$ for the Fermi surface sheet j, $S_{k_z,j}$ is the circumference of the respective Fermi surface sheet j cut at $k_{z,j}$, and $A_{l,k_z,j}$ is the area enclosed by the scattering path length vector trajectory in $\boldsymbol{l}$-space[13] (note that equation (11) as written in Ref.[13] has a typo leading to an erroneous factor of π). The Eq. (17) links the inverse Hall coefficient directly to the band structure of $SrVO_3$, specifically Fermi surface geometry and Fermi velocity, as well as transport relaxation time that is implicit in $l_{av,k_z,j}$ and $A_{l,k_z,j}$. This allows to directly link first principle calculation results shown in Suppl. Chap. 2 to the experimental results. In case of an isotropic, i.e. $\boldsymbol{k}$-independent, transport relaxation time only the geometry of the Fermi surface sheets and Fermi velocity determine the inverse Hall coefficient $(eR_H)^{-1}$ which is obtained from first principles calculation summarized in the Table below, which gives

$$(eR_H)^{-1} = \frac{1}{8\pi^2} \cdot \frac{\left[\int_{-\frac{\pi}{c}}^{\frac{\pi}{c}} l_{av,k_z,1} \cdot S_{k_z,1} \frac{dk_{z,1}}{2\pi} + \int_{-\frac{\pi}{c}}^{\frac{\pi}{c}} l_{av,k_z,2} \cdot S_{k_z,2} \frac{dk_{z,2}}{2\pi} + \int_{-\frac{\pi}{c}}^{\frac{\pi}{c}} l_{av,k_z,3} \cdot S_{k_z,3} \frac{dk_{z,3}}{2\pi}\right]^2}{\int_{-\frac{\pi}{c}}^{\frac{\pi}{c}} A_{l,k_z,1} \frac{dk_{z,1}}{2\pi} + \int_{-\frac{\pi}{c}}^{\frac{\pi}{c}} A_{l,k_z,2} \frac{dk_{z,2}}{2\pi} + \int_{-\frac{\pi}{c}}^{\frac{\pi}{c}} A_{l,k_z,3} \frac{dk_{z,3}}{2\pi}} \qquad (18)$$

$$(eR_H)^{-1} = \frac{1}{8\pi^2} \cdot \frac{\left[0.98 \cdot 10^{23}\text{cm}^{-1} + 1.18 \cdot 10^{23}\text{cm}^{-1} + 2.67 \cdot 10^{23}\text{cm}^{-1}\right]^2}{4.84 \cdot 10^{22}\text{cm} + 4.89 \cdot 10^{22}\text{cm} + 3.69 \cdot 10^{22}\text{cm}} = 2.2 \cdot 10^{22}\text{cm}^{-3} \qquad (19)$$

Suppl. Table S1. Contribution to the inverse Hall coefficient $(eR_H)^{-1}$ for the different Fermi surface sheets of $SrVO_3$.

Fermi surface sheet	$\int \boldsymbol{l_{av,k_z}} \cdot \boldsymbol{S_{k_z}} \frac{\boldsymbol{dk_z}}{\boldsymbol{2\pi}}$ (cm^{-1})	$\int \boldsymbol{A_{l,k_z}} \frac{\boldsymbol{dk_z}}{\boldsymbol{2\pi}}$ (cm)
i	0.98×10^{23}	4.84×10^{22}
ii	1.18×10^{23}	4.89×10^{22}
iii	2.67×10^{23}	3.69×10^{22}

c. Temperature dependence of the Hall coefficient in $SrVO_3$

From Eq. (17) it is evident that in the isotropic limit, i.e. a transport relaxation time $\tau_{\boldsymbol{k}}$ independent of $\boldsymbol{k}$, the inverse Hall coefficient $(eR_H)^{-1}$ is independent of the transport relaxation time: while $l_{av,k_z,j}$ is proportional to $\tau_{\boldsymbol{k}}$ in the numerator of Eq. (17), $A_{k_z,j}$ in the denominator is proportional to $\tau_{\boldsymbol{k}}^{\boldsymbol{2}}$. Indeed, in simple metals $(eR_H)^{-1}$ is found saturate to a temperature-independent value that is only determined by the Fermi surface geometry,[7] which occurs at temperatures higher than about ~0.2-0.3 times the Debye temperature Θ_D. Below this temperature, the transport relaxation time has a $\boldsymbol{k}$-dependence, and thus the Hall coefficient in the weak field limit becomes temperature dependent.

In order to understand the relevance of the temperature dependence of the weak field Hall effect in ultraclean $SrVO_3$ the discussion below is focused on the high and low temperature limits and by taking the three different Fermi surface sheets into account. Note that $SrVO_3$ in the disordered limit has a temperature independent inverse Hall coefficient of about $(eR_H)^{-1} \approx 1.9 \times 10^{22}$ cm^{-3}, which is good agreement with the inverse Hall coefficient calculated for isotropic scattering and also coincidentally in close agreement with

the carrier concentration of 1.8×10^{22} cm^{-3} expected for cubic $SrVO_3$ with a lattice parameter of 3.842 Å and a single electron in the t_{2g} orbitals forming the conduction bands. The temperature-independent Hall coefficient for the disordered limit indicates that the dominant scattering mechanism is temperature independent as well. The origin of this rather trivial temperature dependence occurs if impurity scattering, which is $\boldsymbol{k}$-independent because the likelihood to be scattered at a defect is isotropic, i.e. independent of direction,[13] is the dominant scattering mechanism, overshadowing all other electron scattering phenomena. In contrast, the temperature dependence of the inverse Hall coefficient of ultraclean $SrVO_3$ shows two regimes, a linear regime at room temperature and a quadratic regime at low temperatures.

High temperature: The inverse Hall coefficient of ultraclean samples shows non-saturating linear trend in the ultraclean limit, adding further evidence that the scattering mechanism is not only phononic in nature, indicative of a strange metal behavior. For comparison, in the case of conventional metals like Cu and Ag Dugdale and Firth have shown[16] that with increasing temperature above ~0.2-$0.3\Theta_D$ $(eR_H)^{-1}$ asymptotically approaches 1.40 electrons per unit cell for Cu, and 1.18 electrons per unit cell for Ag, which is within a few percent error of the calculated value obtained by taking their respective band structures into account. The linear trend in $(eR_H)^{-1}$ occurred above 100 K coincides with 0.2-0.3 of the Debye temperature of $SrVO_3$ (Θ_D~350 K).[17,18] The behavior of a linear non-saturating inverse Hall coefficient has been observed in the strange metal phase of the cuprates as well.

Low temperature: For ultraclean samples at low temperatures the dominant transport scattering process is neither from impurity scattering due to the low defect concentration, nor phonon scattering, which if large momentum phonon states are populated at elevated temperature, allows for electron scattering with large momentum transfer and thus electron scattering across the entire Brillouin zone, therefore averaging out the intricacies of the Fermi surface sheets. In absence of these scattering phenomena, the local shape and properties of Fermi surface sheets can give rise to an additional temperature dependence. The quadratic increase of the inverse Hall coefficient found for $SrVO_3$ in the ultraclean limit at low temperatures $T<<\Theta_D$ is attributed to a more pronounced $\boldsymbol{k}$-dependence of the transport relaxation times dominated by electron-electron scattering times, which otherwise is averaged out and therefore hidden. Generally, according to Eq. (17) any deviation from a $\boldsymbol{k}$-independent scattering mechanism with temperature will result in a temperature dependence of the inverse Hall coefficient: the sum of the individual average scattering path length vector $l_{av,k_z,j}$ (proportional to τ_k) is squared in the numerator, while the sum of the square values of the scattering path length vector $l_{av,k_z,j}$ (implicit in the area A_l) occurs in the denominator.

At low enough temperatures phonon scattering becomes more pronounced for $\boldsymbol{k}$-states in the proximity of large curvatures,[19,20] which decreases the transport relaxation times thus reducing their scattering path length vector. In the case of $SrVO_3$ the outer Fermi surface sheet *iii* has the largest curvature where the 'jungle-gym' surface sheets meet. Therefore, it is expected to have shorter relaxation times relative to the $\boldsymbol{k}$-states of the inner Fermi surface sheets *i* and *ii*. Therefore, upon reducing the temperature the total relaxation time of states in the outermost Fermi surface sheet *iii* decreases relative to the inner lying electron-like Fermi surface sheets. Although quite counterintuitive, the associated reduction of hole mobility gives rise to a more pronounced hole-like character and therefore causing a nonlinear Hall effect in the transient temperature regime. Specifically, from Suppl. Fig. S8 it can be seen that for electron and hole carriers with $|N_1/N_2|=2$ and $\mu_1/\mu_2=1$ the nonlinearity of the Hall effect is negligible, but becomes more pronounced if the majority carrier type enhanced the mobility, i.e. $|N_1/N_2|=2$ and $\mu_1/\mu_2=4$.

As discussed in the main text the simplest approximation to explain the measured temperature dependence using Eq. (17) is to assume a different, i.e. shorter, transport relaxation time for the outermost Fermi surface sheet *iii*. Taking τ_i for the inner Fermi surface sheets *i* and *ii*, and τ_o for the outer 'jungle gym'

Fermi surface *iii*, the transport relaxation time ratio $\zeta = \tau_o/\tau_i$ reduces the inverse Hall coefficient determined from the geometric analysis

$$(eR_H)^{-1} = \frac{1}{8\pi^2} \cdot \frac{\left[0.98\cdot 10^{23}\mathrm{cm}^{-1} + 1.18\cdot 10^{23}\mathrm{cm}^{-1} + \zeta\cdot 2.67\cdot 10^{23}\mathrm{cm}^{-1}\right]^2}{4.84\cdot 10^{22}\mathrm{cm} + 4.89\cdot 10^{22}\mathrm{cm} + \zeta^2\cdot 3.69\cdot 10^{22}\mathrm{cm}} \quad (20)$$

The inverse Hall coefficient reduces from the value obtained for isotropic scattering only if the transport relaxation time ratio is smaller than unity. Since isotropic scattering for the ultraclean sample is best approximated at room temperature, the occurrence of a nonlinear Hall effect with majority carrier electrons having a higher mobility indicates that the relative contribution of hole like transport increased with decreasing temperature, suggesting that the transport relaxation time of electrons increased much more compared to holes at low temperatures.

Supplemental Materials References

1. Brahlek, M. *et al.* Frontiers in the Growth of Complex Oxide Thin Films: Past, Present, and Future of Hybrid MBE. *Advanced Functional Materials* **28**, 1702772 (2017).
2. Moyer, J. A., Eaton, C. & Engel-Herbert, R. Highly Conductive SrVO3 as a bottom electrode for functional perovskite oxides. *Advanced materials (Deerfield Beach, Fla.)* **25**, 3578–82 (2013).
3. Brahlek, M., Zhang, L., Eaton, C., Zhang, H.-T. & Engel-Herbert, R. Accessing a growth window for SrVO3 thin films. *Applied Physics Letters* **107**, 143108 (2015).
4. Brahlek, M. *et al.* Mapping growth windows in quaternary perovskite oxide systems by hybrid molecular beam epitaxy. *Applied Physics Letters* **109**, 101903 (2016).
5. Schroder, D. K. *Semiconductor Material and Device Characterization*. (Wiley-IEEE Press, 2015).
6. Pippard, A. B. *Magnetoresistance in Metals*. (Cambridge University Press, 1989).
7. Hurd, C. M. *The Hall Effect in Metals and Alloys*. (Plenum Press, 1972). doi:10.1007/978-1-4757-0465-5.
8. Shoenberg, D. *Magnetic Oscillations in Metals*. (Cambridge University Press, 1984).
9. Inoue, I., Bergemann, C., Hase, I. & Julian, S. Fermi Surface of 3d1 Perovskite CaVO3 near the Mott Transition. *Physical Review Letters* **88**, 236403 (2002).
10. Haule, K., Yee, C.-H. & Kim, K. Dynamical mean-field theory within the full-potential methods: Electronic structure of CeIrIn5, CeCoIn5, and CeRhIn5. *Physical Review B* **81**, 195107 (2010).
11. Haule, K. & Birol, T. Free Energy from Stationary Implementation of the DFT + DMFT Functional. *Physical Review Letters* **115**, 256402 (2015).
12. Paul, A. & Birol, T. Strain tuning of plasma frequency in vanadate, niobate, and molybdate perovskite oxides. *Physical Review Materials* **3**, 85001 (2019).
13. Ong, N. P. Geometric interpretation of the weak-field Hall conductivity in two-dimensional metals with arbitrary Fermi surface. *Physical Review B* **43**, 193–201 (1991).
14. Fert, A. Transport in magnetic alloys: Scattering asymmetries (anisotropic scattering, skew scattering, side-jump). *Physica B+C* **86–88**, 491–500 (1977).
15. Rhyne, J. J. Anomalous and Ordinary Hall Effect in Terbium. *Journal of Applied Physics* **40**, 1001–1003 (1969).
16. Dugdale, J. S. & Firth, L. D. The Hall coefficient of dilute alloys of copper and silver. *Journal of Physics C: Solid State Physics* **2**, 319 (1969).
17. Maekawa, T., Kurosaki, K. & Yamanaka, S. Physical properties of polycrystalline SrVO3−δ. *Journal of Alloys and Compounds* **426**, 46–50 (2006).
18. Parveen, A. & Gaur, N. K. Elastic and thermodynamic properties of AVO3 (A=Sr, Pb) perovskites. *Physica B: Condensed Matter* **407**, 500–504 (2012).
19. Ziman, J. M. Approximate Calculation of the Anisotropy of the Relaxation Time of the Conduction Electrons in the Noble Metals. *Physical Review* **121**, 1320–1324 (1961).

20. Ziman, J. M. *Electrons and Phonons*. *Electrons and Phonons* (2007). doi:10.1093/acprof:oso/9780198507796.001.0001.
